# Supplementary material for: Brine residues and organics in the Urvara basin on Ceres
Source: Nat Commun. 2022 Feb 22;13:927. doi: 10.1038/s41467-022-28570-8 (PMC8863799; doi:10.1038/s41467-022-28570-8)
Supplement: Supplementary file 1 — Supplementary Information [file 41467_2022_28570_MOESM1_ESM.pdf]

Supplementary information

The Urvara basin on Ceres – brine residues and organics

A. Nathues[1*], M. Hoffmann[1], N. Schmedemann[2], R. Sakar[1], G. Thangjam[3], K. Mengel[1], J. Hernandez[1], H. Hiesinger[2], and J.H. Pasckert[2]

[1]Max Planck Institute for Solar System Research, Justus-von-Liebig-Weg 3, 37077 Goettingen, Germany; [2]Institut für Planetologie, WWU Münster, Germany; [3]School of Earth and Planetary Sciences, National Institute of Science Education and Research, NISER, HBNI, Bhubaneswar, India

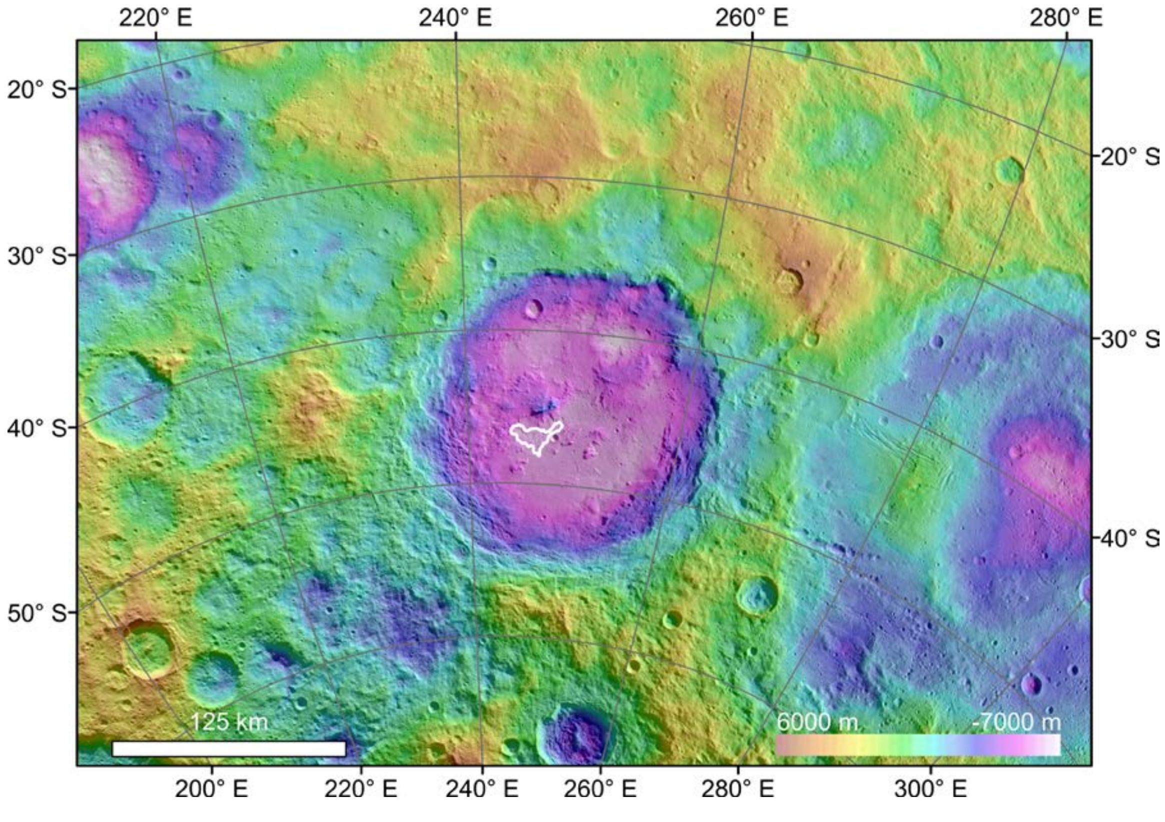

Supplementary Figure 1: Topographic map of Urvara and its vicinity. View extracted from the global low altitude mapping orbit shape mode [1]. The basin exhibits a central ridge that rises ~3 km above the southern floor. The well-preserved northern crater wall rises up to ~6–7 km above the northern floor. The low-lying area south of the central ridge (polygon) shows a “pitted texture” (see main text and Supplementary Fig. 2).

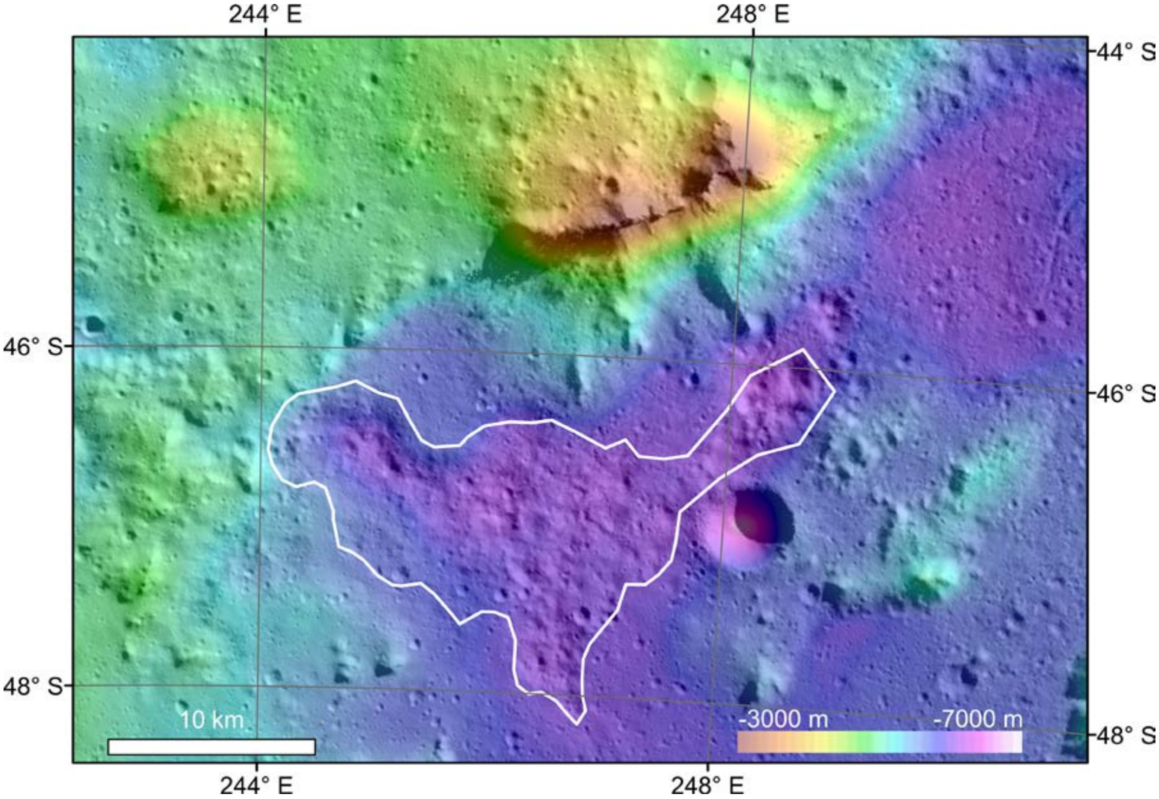

Supplementary Figure 2: Topographic map of the central peak and the "pitted area". The displayed figure is a combination of colour-coded topographic information derived from our low altitude mapping orbit (LAMO) digital terrain model generated with the Ames Stereo Pipeline [2] and a translucent clear filter mosaic, both obtained from LAMO orbit data. The polygon marks a low-lying floor area hosting potential sublimation or evaporation pits.

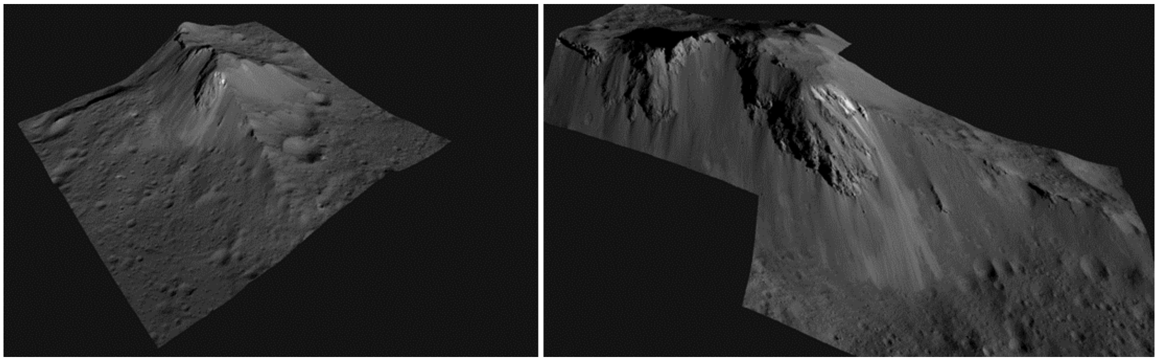

Supplementary Figure 3: Perspective views of the central ridge in clear filter. The left view is at a pixel scale of ~10–15 m (displayed mosaic size is about 22 by 15 km), while the right view is at a pixel scale of ~5 m (displayed mosaic size is about 15 by 4 km). The brightest material on the central ridge is found near its summit at one of the main cliffs in the north-east. Here bright material moves downslope and brightens dark ridge material. The view is towards the west (left image) and north-west (right image).

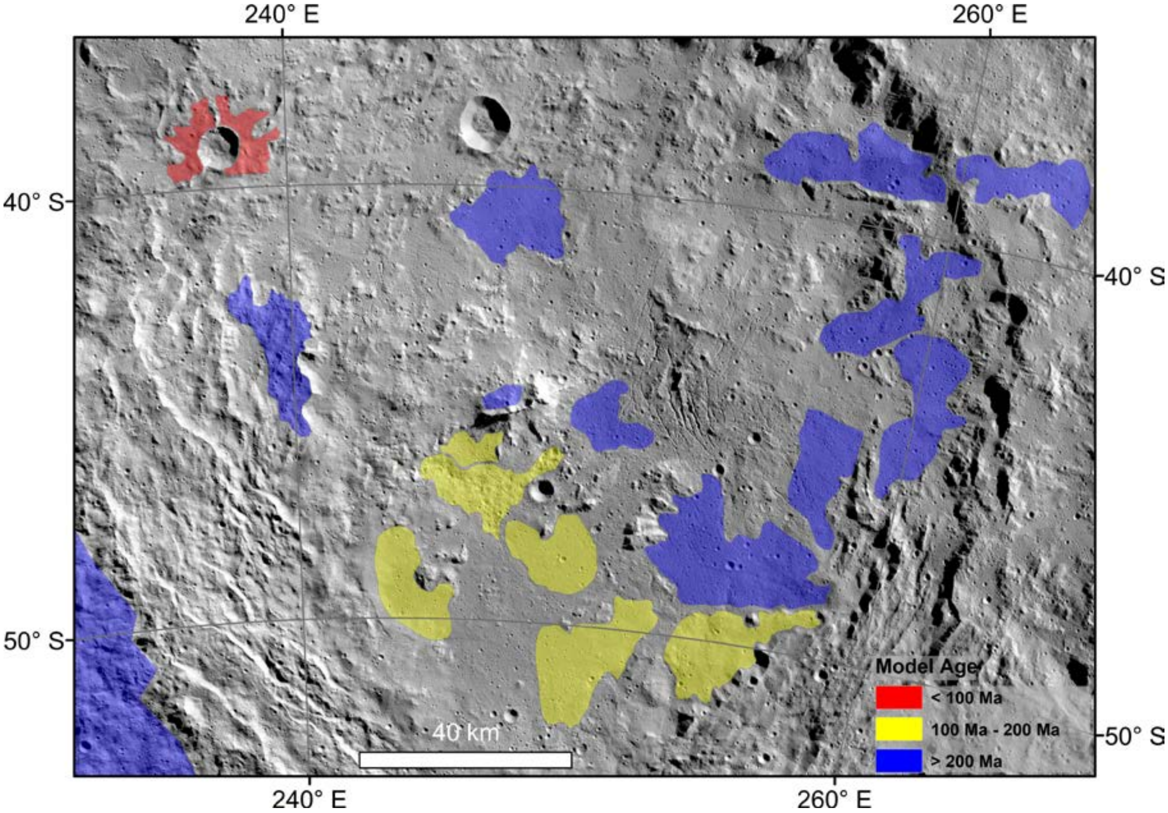

Supplementary Figure 4: Overview of the major age units. Three major age periods are identified. See Fig. 10 for labels.

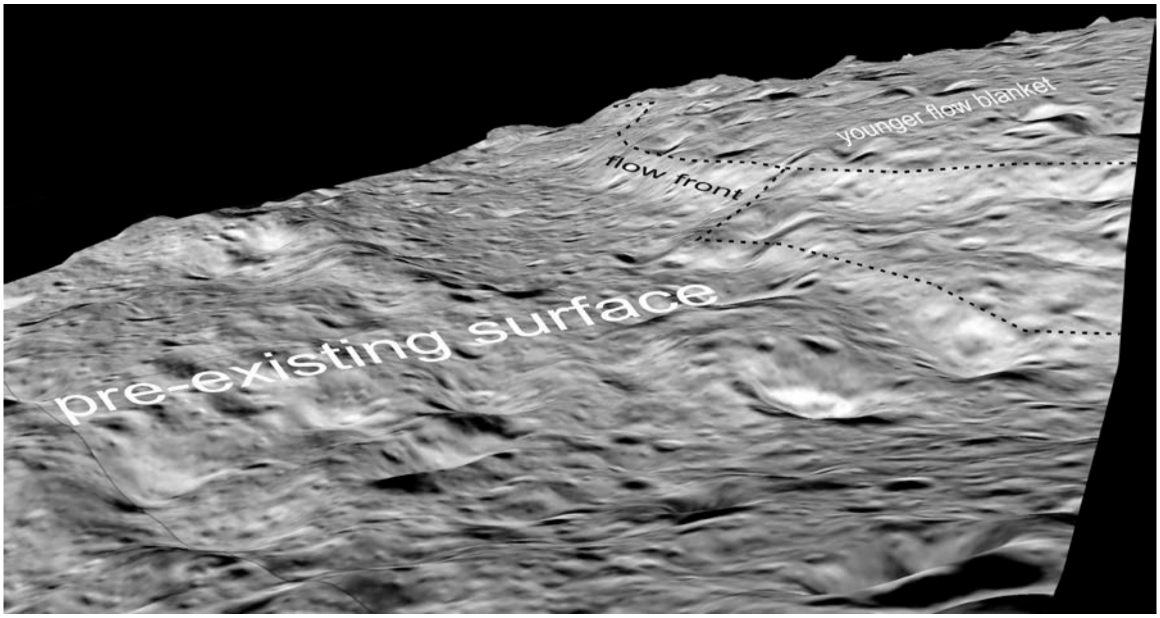

Supplementary Figure 5: Perspective view of a flow front on the smooth material looking south-east. The younger smooth material ("younger flow blanket") is seen in the

upper right corner of the image, the older smooth material (“pre-existing surface”) between the centre and lower left corner. A distinct 60 to 80 m high flow front, which separates the younger from the old smooth material, overtops the former surface. Imagery is draped over a topography derived from a stereo pair of images (FC21C0098557 and FC21C0098253) with a pixel scale of ~5 m from the extended mission phase 2 orbit. The view is exaggerated two times. The detail is located on the southern floor between counting Areas 1A and 8.

Grooves on Urvara’s floor. Grooves, being further indicators of surface mobility, cluster on the eastern floor mainly in two regions: the larger ones are located near the centre of the crater (Supplementary Fig. 6a/f), while the others are found south-east of the central ridge (Supplementary Fig. 6a/b). High-resolution imagery reveals that the grooves on the south-eastern floor consist of merged small (~100 m diameter), linearly oriented, subcircular pits lacking raised rims (Supplementary Fig. 6b). These grooves are up to ~100 m wide and transect the boundary between the two geologic units Ufs and UYs (for units see Fig. 3 in [3]). Thus, these grooves postdate the emplacement of these surface units. The grooves east of the central peak (Supplementary Fig. 6a/f) are up to ~600–700 m wide and ~1 km deep and thus significantly larger; they exhibit an anastomosing/inosculating pattern (Supplementary Fig. 6c). Similarly to the smaller grooves (Supplementary Fig. 6a/b), these larger grooves display subcircular to elliptical wall segments, indicating a formation due to many merging “collapse pits” (Supplementary Fig. 6c). There are no indications of raised pit rims or any signs of levees in the grooves. At several locations, calving of the inner walls of the grooves occur, which might indicate the process by which they widen. Some of the grooves, particularly the larger ones, display smooth/rounded rims, in contrast to the smaller grooves in the same area, which exhibit sharp rims and appear fresh (Supplementary Fig. 6c and 6d), suggesting a different formation age. The large grooves taper out towards the south, but further smaller grooves traversing the region between the larger grooves and the smaller grooves in the south-east might suggest a link between them. In addition to the north-south-oriented grooves, there is another set of north-east-south-west-oriented grooves, which appear less prominent (see Supplementary Fig. 6e). Establishing a clear age relationship between the intersecting grooves is not possible because of the absence of cross-cutting relationships. Nonetheless, the subdued topographic expression and rounded edges of these less prominent grooves might indicate that these are older. However, the north-east-south-west-oriented grooves display the same basic nature, linear chains of linked circular pits without raised rims (Supplementary Fig. 6e). The larger grooves, east of the central ridge, are intersected by a distinct set of arcuate grooves that do not show association with pit craters; rather these appear more like faults (see Supplementary Fig. 6f). These grooves (or arcuate faults) are shallower and older than the north-south-

oriented grooves as inferred from their cross-cutting relationship. Grooves on the western floor also originate from linearly merged collapse pits (Supplementary Fig. 6g). However, here the pits, and thereby the grooves, have a fresher appearance than the grooves on the eastern floor. A reason for the difference in apparent freshness may be due to different consolidation, since the western floor is characterized by a rougher topography.

Grooves (or linear depressions) on the floor of Urvara are linkages of linearly oriented, subcircular to elliptical pits lacking raised rims, which do not seem to be caused by sublimation or devolatilization processes reported elsewhere on Ceres, and are suspected to be present at another locality in Urvara (see the section "Geology of Urvara basin" and Supplementary Fig. 2). These pits rather are akin to pit crater chains, i.e. caused by surface collapses [4]. Linearly oriented pits have formed at several locations on Urvara's floor and it seems that they belong to more than one generation. Several formation mechanisms for pit crater chains have been described in the literature. Most are the result of underground voids and the subsequent collapse of the surface. Mechanisms are dilational normal faulting [4, 5], collapsed lava tubes [6], collapsed magma chambers [7, 8], and dissolution and removal of subsurface materials [9]. Alternatively, a viscous layer underneath Urvara's floor could explain the floor grooves [10, 11]. By virtue of the low elevation of the floor, Urvara could be very close to the frictional-to-viscous transition zone [10]. The overburdening pressure of the upper layer of material would initiate ductile flow in the viscous layer, which in turn would generate stresses in the overlying brittle material, thereby resulting in graben formation [11]. The viscous relaxation could be made viable either through the warm, mid-latitude temperatures [12] or through the impact-generated heat itself [13]. The grooves are certainly not secondary crater chains, as we do not observe raised rims or ejecta deposits. Their appearance is also inconsistent with sublimation or devolatilization pits, which would rather tend to form irregular clusters covering wider areas and not linear chains. Their most plausible cause of origin is the movement of smooth material on large-scale slopes across the floor.

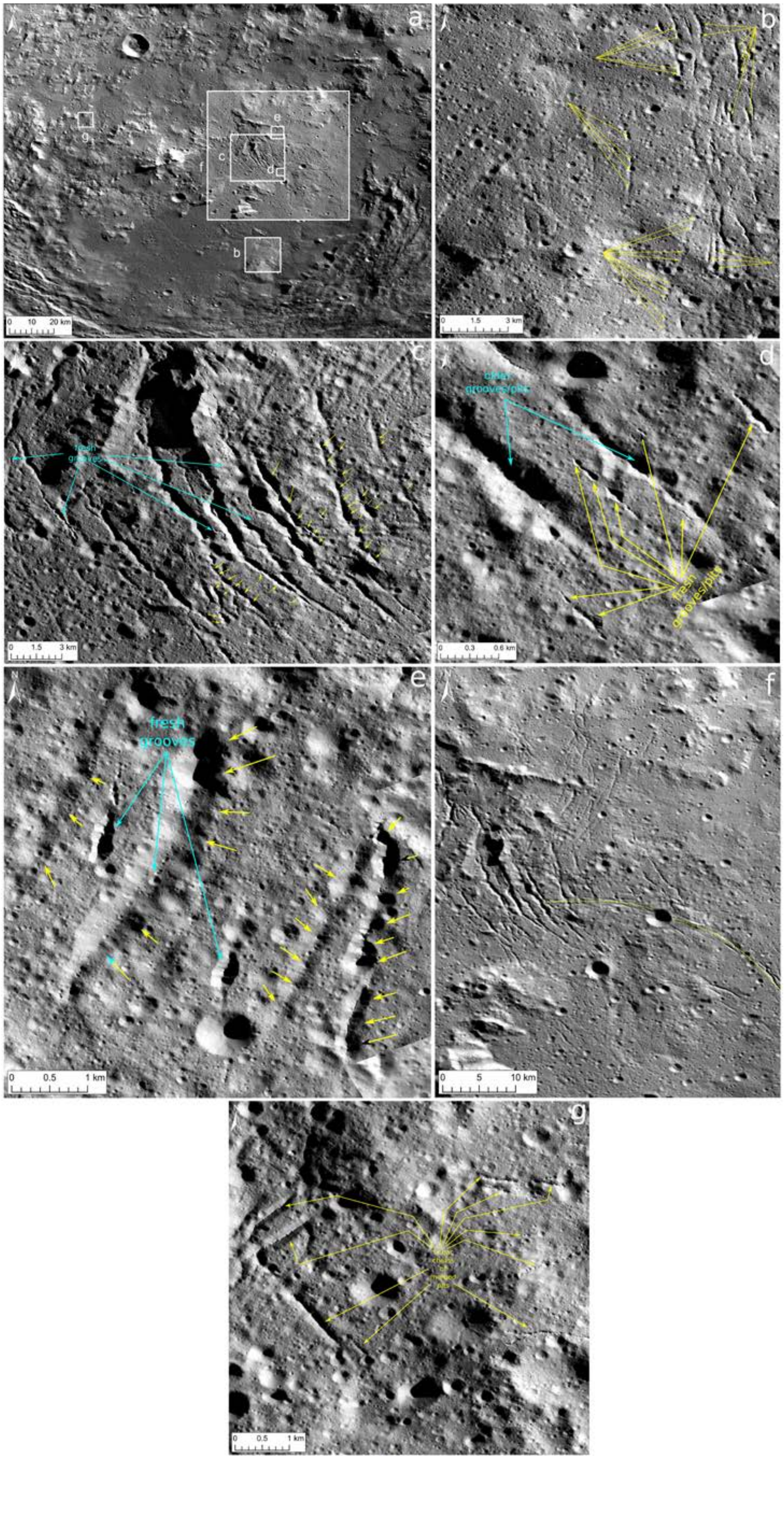

a
g
e
c
d
f
b
0 10 20 km
0 1.5 3 km
fresh grooves
0 1.5 3 km
fresh grooves/pits
0 0.3 0.6 km
fresh grooves
0 0.5 1 km
0 5 10 km
0 0.5 1 km

Supplementary Figure 6: Different groups of grooves traverse the floor of Urvara. (a) Overview of the locations of the different groups. (b) North-south-oriented small grooves located south-east of the central ridge composed of linearly oriented pits. (c) Larger grooves east of the central ridge, with a few fresh-appearing grooves; these grooves display subcircular to elliptical wall segments. (d) A closer look at some fresher grooves, forming between older grooves. (e) North-east-south-west-oriented intersecting smaller grooves near the grooves in (d). These grooves are also composed of circular pits without raised rims. (f) Arcuate grooves, interpreted as faults, intersecting the grooves on the eastern floor of Urvara. These grooves do not show an association with pits. (g) Grooves on the western floor in rugged terrain.

Supplementary Information Table 1: Model ages and respective errors of analysed areas.

Area Name	Area [km²]	Age [Myr]	Age "+" Error [Myr]	Age "-" Error [Myr]
1A	300.9	160	10	10
1B	254.2	160	10	10
2A	291.7	250	20	20
2B	182.9	240	30	30
3	259.1	240	20	20
4A	233.5	290	30	30
4B	284.8	280	20	20
4C	206.8	270	30	30
5	187.1	150	30	30
6	179.0	150	20	20
7	218.9	240	20	20
8	480.7	280	20	20
9	26.0	220	100	90
10	51.2	140	30	30
11 (background)	0.47	170	90	70
11 (resurfacing)	0.47	21	8	8
13	121.2	270	40	40
14	155	7.9	1	1
15	189.7	150	20	20
16	442.7	260	20	20
17	4103	240	10	10

Supplementary Information Table 2: Crater plots and areas of measurement
(numbers in crater plots are rounded to two significant figures).

Area 1A

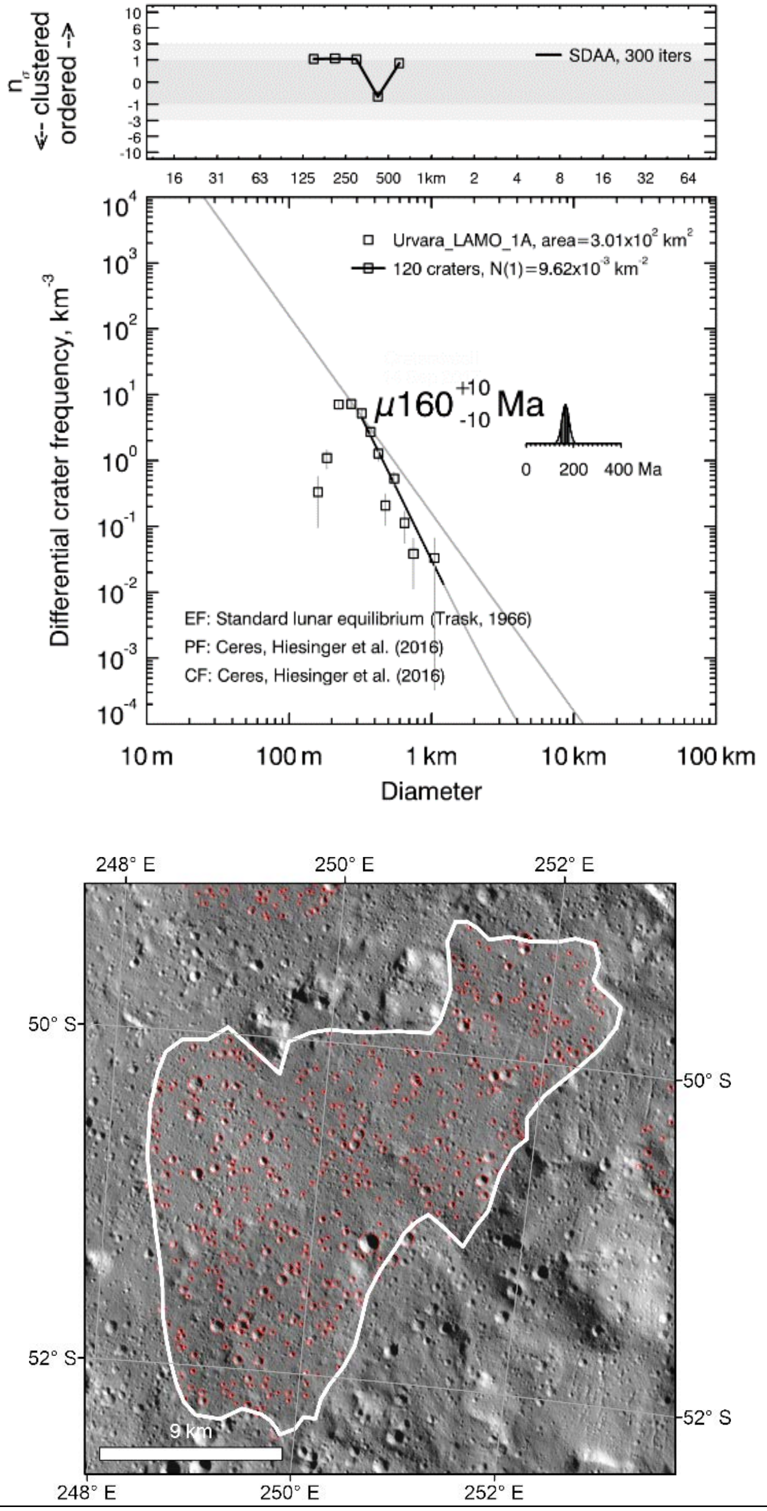

n_σ
<-- clustered
ordered -->
SDAA, 300 iters
16 31 63 125 250 500 1km 2 4 8 16 32 64
Urvara_LAMO_1A, area=3.01x10^2 km^2
120 craters, N(1)=9.62x10^-3 km^-2
$\mu 160^{+10}_{-10}$ Ma
0 200 400 Ma
Differential crater frequency, km^-3
EF: Standard lunar equilibrium (Trask, 1966)
PF: Ceres, Hiesinger et al. (2016)
CF: Ceres, Hiesinger et al. (2016)
10 m 100 m 1 km 10 km 100 km
Diameter
248° E
250° E
252° E
50° S
52° S
9 km

Area 1B

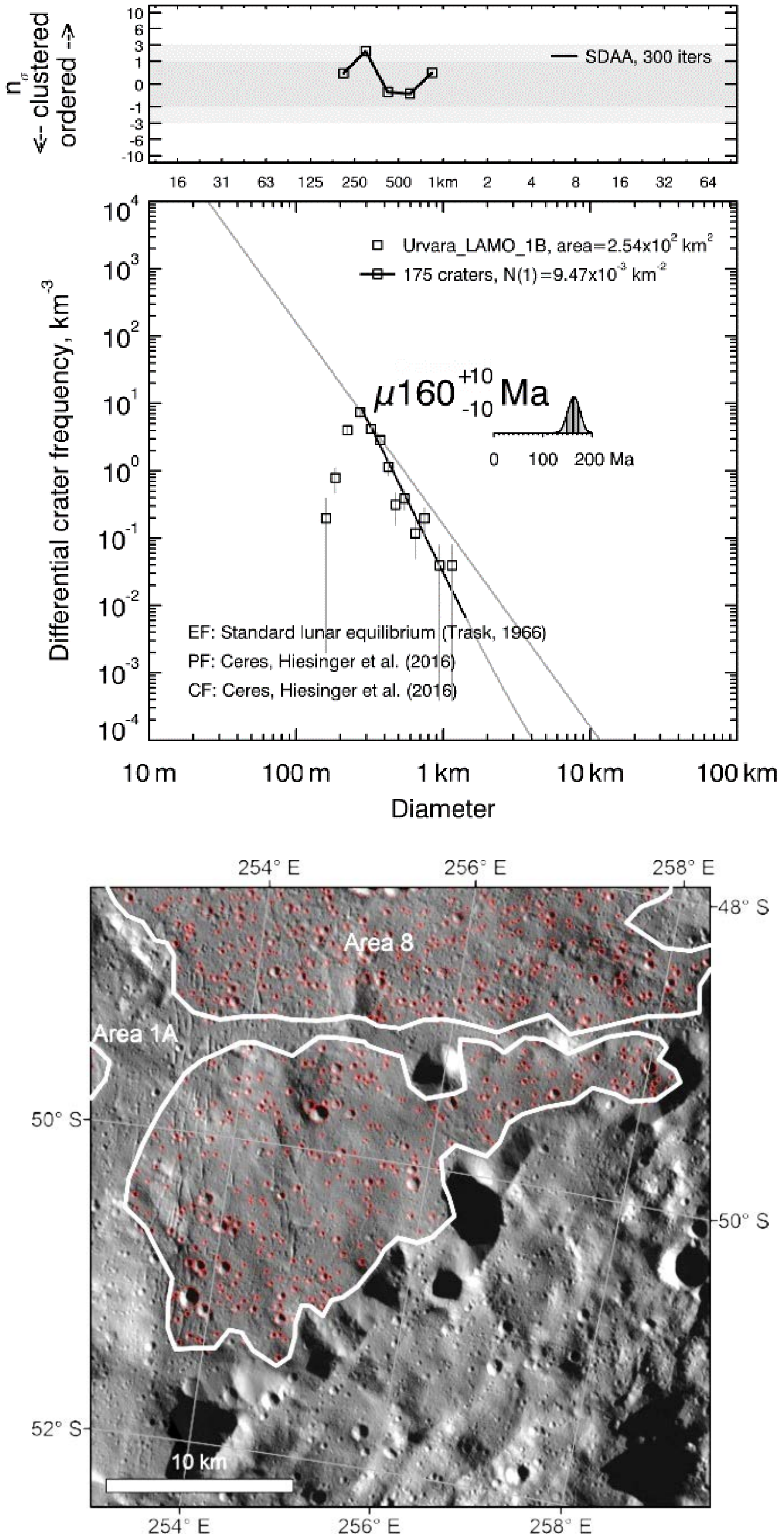

n_σ
<-- clustered
ordered -->
SDAA, 300 iters
Urvara_LAMO_1B, area=2.54x10^2 km^2
175 craters, N(1)=9.47x10^-3 km^-2
μ160 +10 -10 Ma
0 100 200 Ma
EF: Standard lunar equilibrium (Trask, 1966)
PF: Ceres, Hiesinger et al. (2016)
CF: Ceres, Hiesinger et al. (2016)
Differential crater frequency, km^-3
Diameter
10 m
100 m
1 km
10 km
100 km
Area 8
Area 1A
254° E
256° E
258° E
48° S
50° S
52° S
10 km

Area 2A

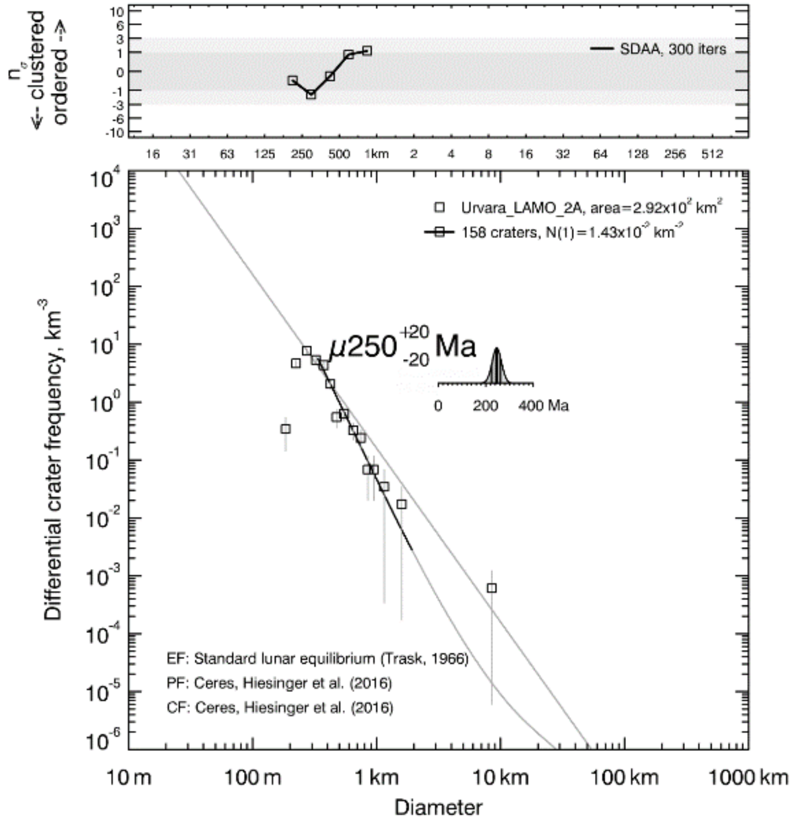

SDAA, 300 iters
Urvara_LAMO_2A, area=2.92x10^2 km^2
158 craters, N(1)=1.43x10^-2 km^-2
μ250 +20 -20 Ma
0 200 400 Ma
EF: Standard lunar equilibrium (Trask, 1966)
PF: Ceres, Hiesinger et al. (2016)
CF: Ceres, Hiesinger et al. (2016)
Differential crater frequency, km^-3
Diameter

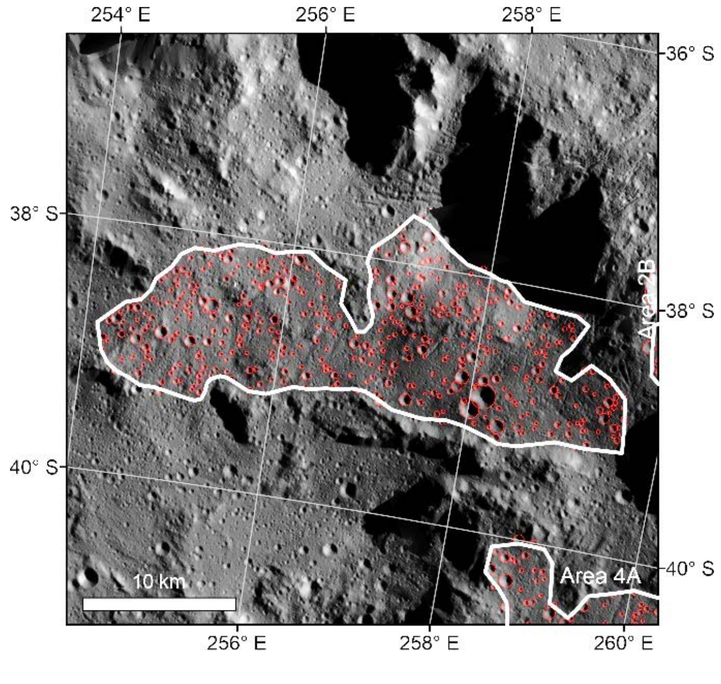

254° E
256° E
258° E
36° S
38° S
40° S
260° E
Area 2B
Area 4A
10 km

Area 2B

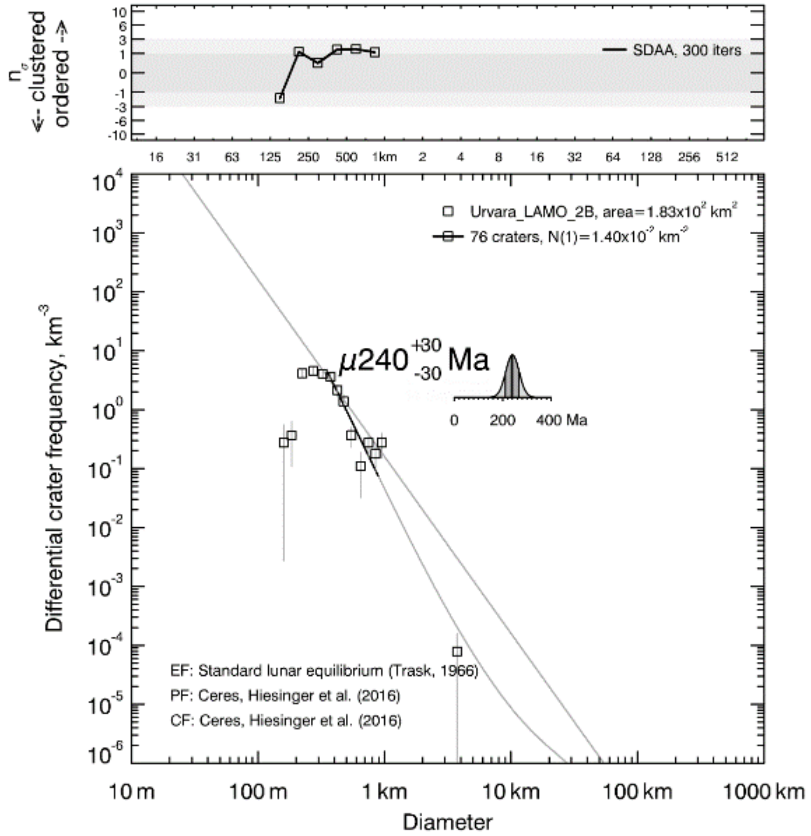

n_σ
<-- clustered
ordered -->
SDAA, 300 iters
16 31 63 125 250 500 1km 2 4 8 16 32 64 128 256 512
Urvara_LAMO_2B, area=1.83x10^2 km^2
76 craters, N(1)=1.40x10^-2 km^-2
μ240 +30 -30 Ma
0 200 400 Ma
Differential crater frequency, km^-3
EF: Standard lunar equilibrium (Trask, 1966)
PF: Ceres, Hiesinger et al. (2016)
CF: Ceres, Hiesinger et al. (2016)
10 m 100 m 1 km 10 km 100 km 1000 km
Diameter

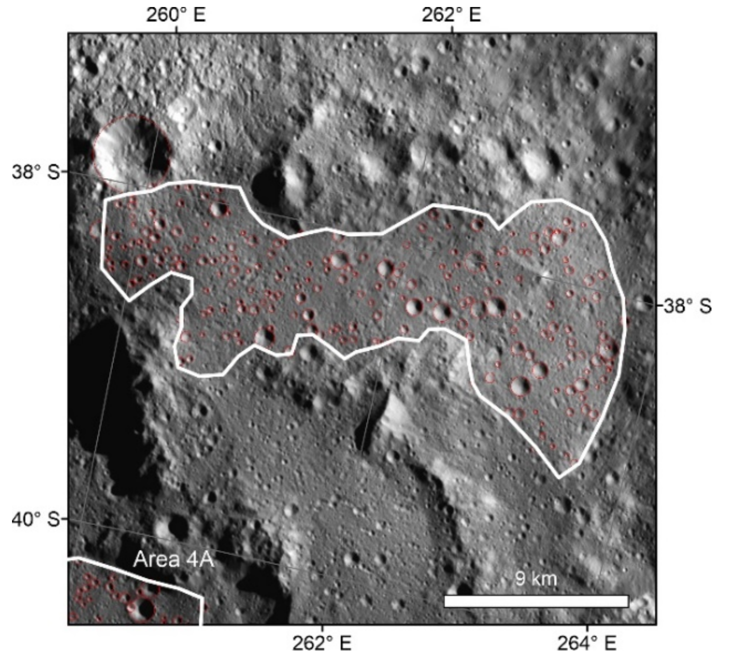

260° E
262° E
38° S
38° S
40° S
Area 4A
9 km
262° E
264° E

Area 3

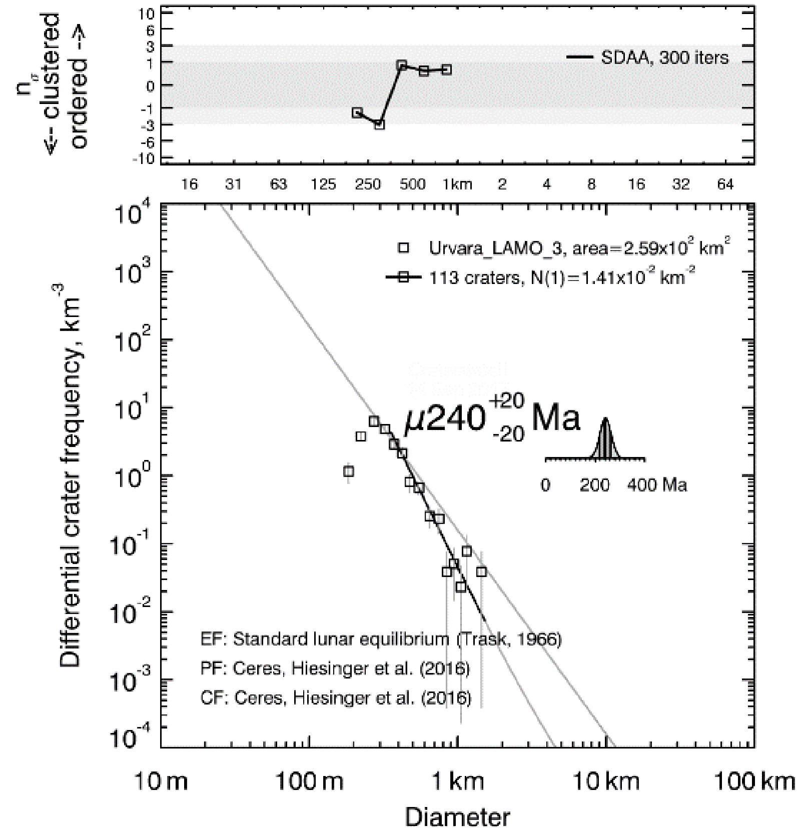

n_σ
<-- clustered
ordered -->
SDAA, 300 iters
16 31 63 125 250 500 1km 2 4 8 16 32 64
Urvara_LAMO_3, area=2.59x10^2 km^2
113 craters, N(1)=1.41x10^-2 km^-2
μ240 +20 -20 Ma
0 200 400 Ma
EF: Standard lunar equilibrium (Trask, 1966)
PF: Ceres, Hiesinger et al. (2016)
CF: Ceres, Hiesinger et al. (2016)
Differential crater frequency, km^-3
10 m
100 m
1 km
10 km
100 km
Diameter

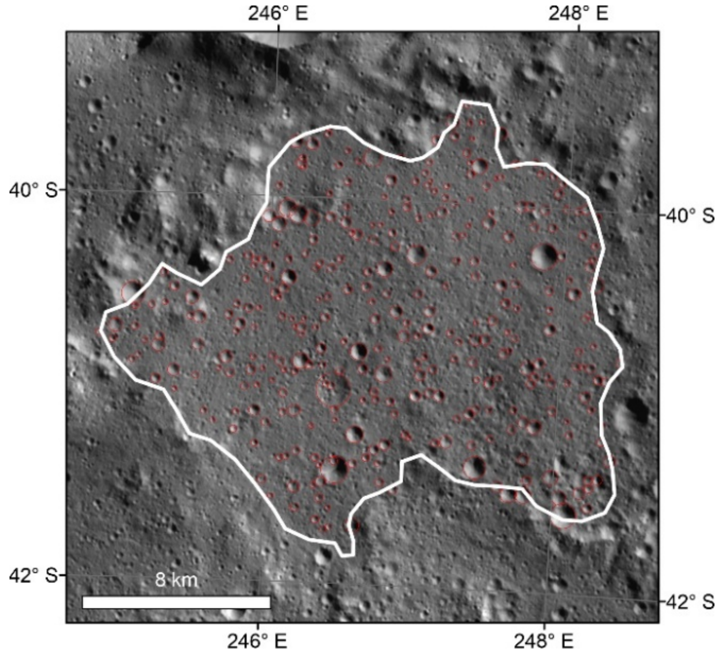

246° E
248° E
40° S
40° S
42° S
42° S
8 km
246° E
248° E

Area 4A

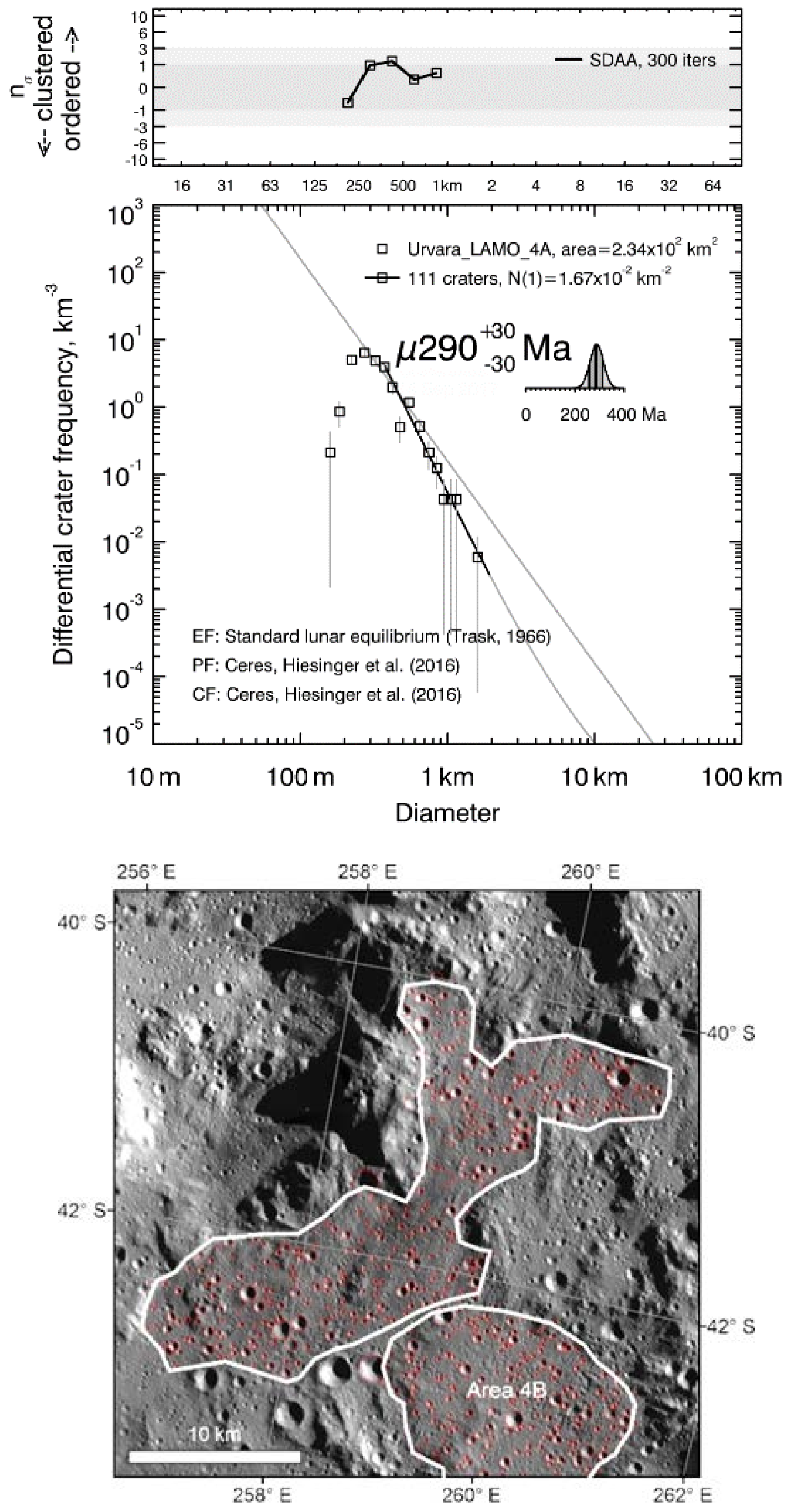

n_σ
<-- clustered
ordered -->
10
6
3
1
0
-1
-3
-6
-10
SDAA, 300 iters
16
31
63
125
250
500
1km
2
4
8
16
32
64
Differential crater frequency, km^-3
Urvara_LAMO_4A, area=2.34x10^2 km^2
111 craters, N(1)=1.67x10^-2 km^-2
μ290 +30 -30 Ma
0
200
400 Ma
EF: Standard lunar equilibrium (Trask, 1966)
PF: Ceres, Hiesinger et al. (2016)
CF: Ceres, Hiesinger et al. (2016)
10 m
100 m
1 km
10 km
100 km
Diameter
256° E
258° E
260° E
40° S
42° S
40° S
42° S
Area 4B
10 km
258° E
260° E
262° E

Area 4B

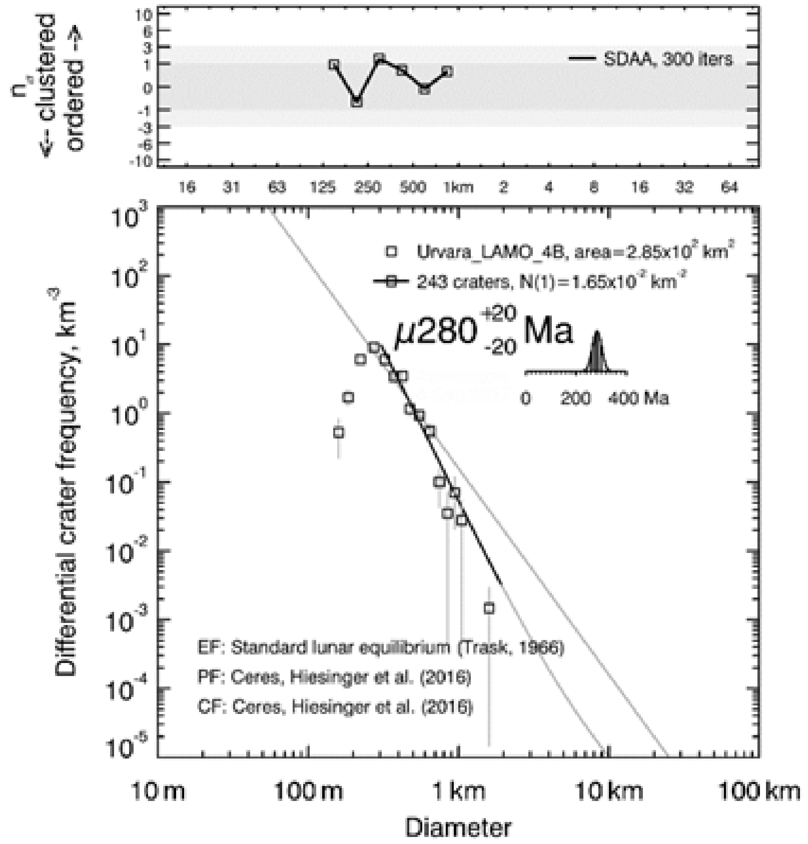

n_σ
<-- clustered
ordered -->
SDAA, 300 iters
Differential crater frequency, km^-3
Urvara_LAMO_4B, area=2.85x10^2 km^2
243 craters, N(1)=1.65x10^-2 km^-2
μ280 +20 -20 Ma
0 200 400 Ma
EF: Standard lunar equilibrium (Trask, 1966)
PF: Ceres, Hiesinger et al. (2016)
CF: Ceres, Hiesinger et al. (2016)
10 m
100 m
1 km
10 km
100 km
Diameter

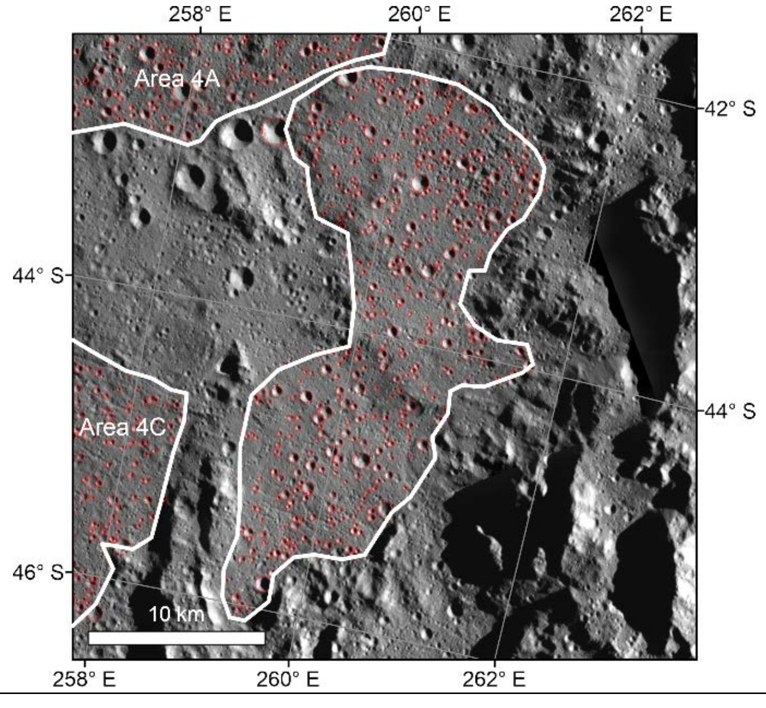

258° E
260° E
262° E
42° S
44° S
44° S
46° S
Area 4A
Area 4C
10 km
258° E
260° E
262° E

Area 4C

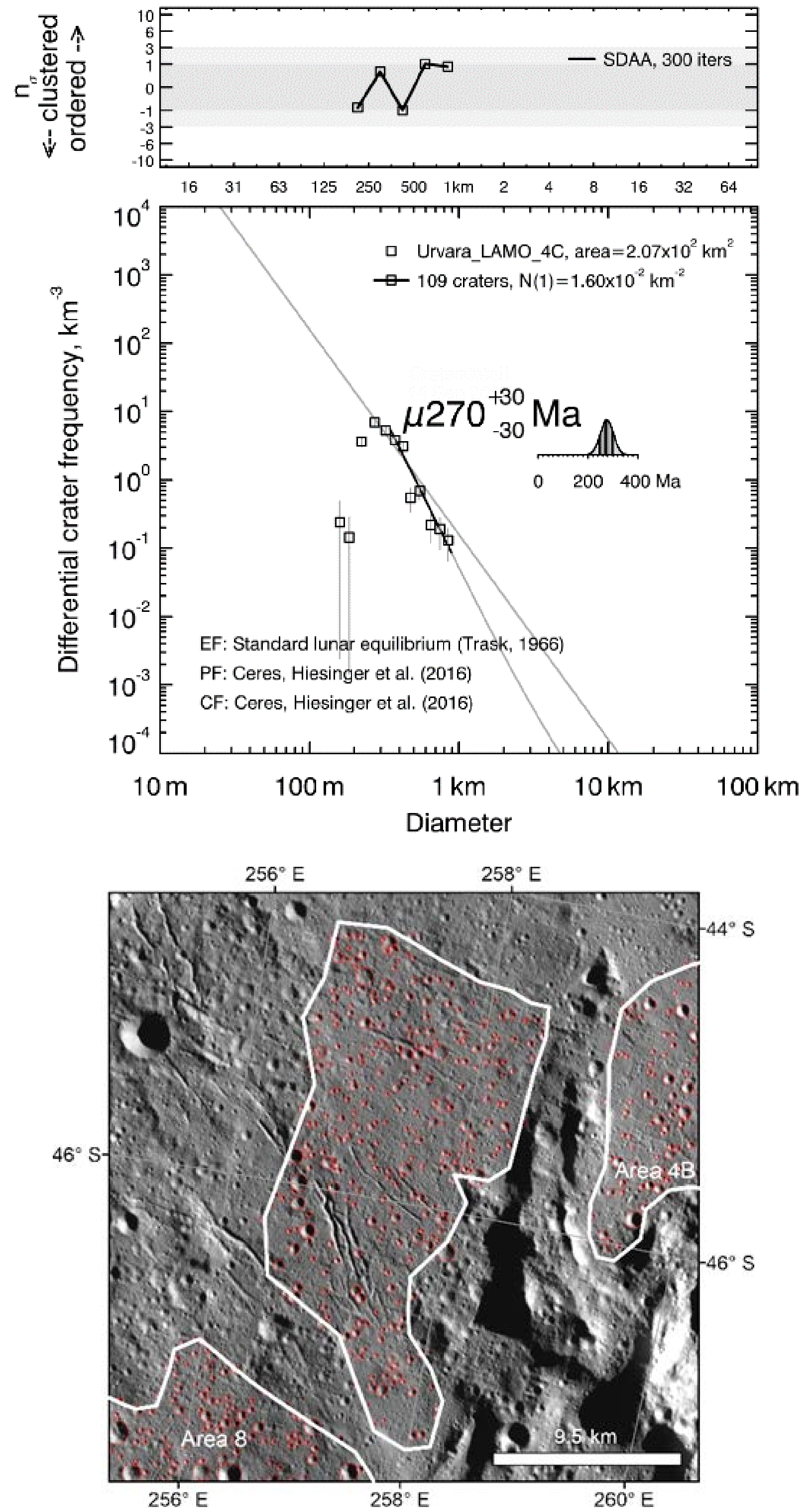

n_σ
<-- clustered
ordered -->
SDAA, 300 iters
16 31 63 125 250 500 1km 2 4 8 16 32 64
Urvara_LAMO_4C, area=2.07x10^2 km^2
109 craters, N(1)=1.60x10^{-2} km^{-2}
$\mu 270^{+30}_{-30}$ Ma
0 200 400 Ma
Differential crater frequency, km^{-3}
EF: Standard lunar equilibrium (Trask, 1966)
PF: Ceres, Hiesinger et al. (2016)
CF: Ceres, Hiesinger et al. (2016)
10 m 100 m 1 km 10 km 100 km
Diameter
256° E
258° E
260° E
44° S
46° S
Area 4B
Area 8
9.5 km

Area 5

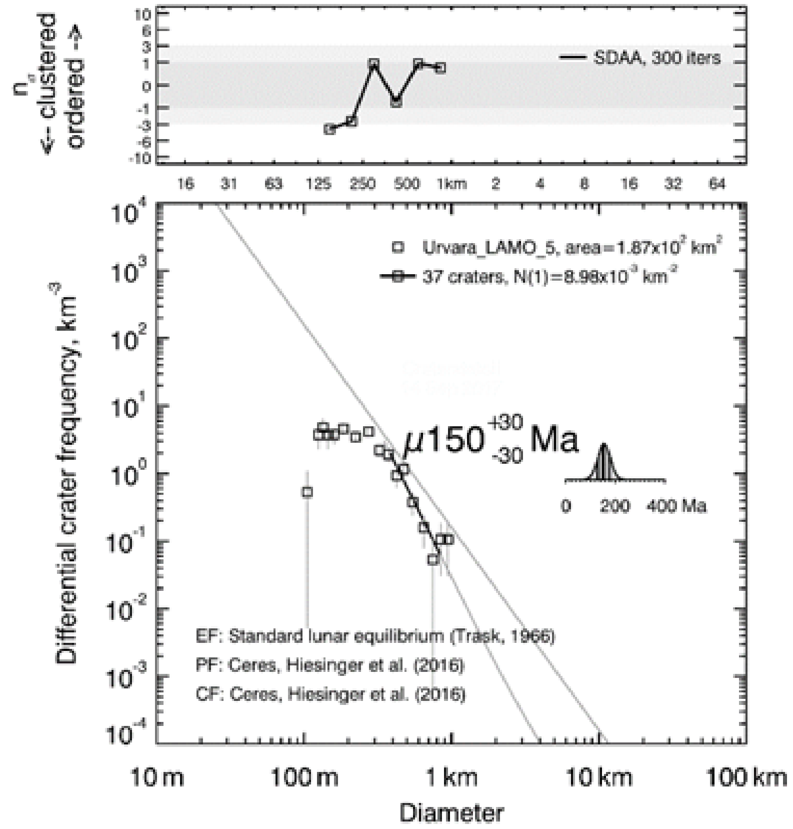

n
<-- clustered
ordered -->
SDAA, 300 iters
Urvara_LAMO_5, area=1.87x10^2 km^2
37 craters, N(1)=8.98x10^-3 km^-2
μ150 +30 -30 Ma
0 200 400 Ma
EF: Standard lunar equilibrium (Trask, 1966)
PF: Ceres, Hiesinger et al. (2016)
CF: Ceres, Hiesinger et al. (2016)
Differential crater frequency, km^-3
10 m
100 m
1 km
10 km
100 km
Diameter

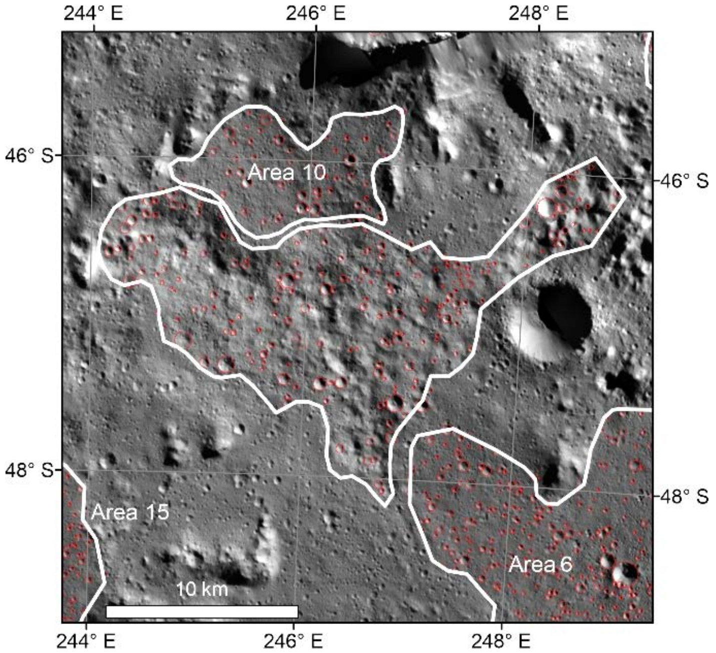

244° E
246° E
248° E
46° S
48° S
Area 10
Area 15
Area 6
10 km

Area 6

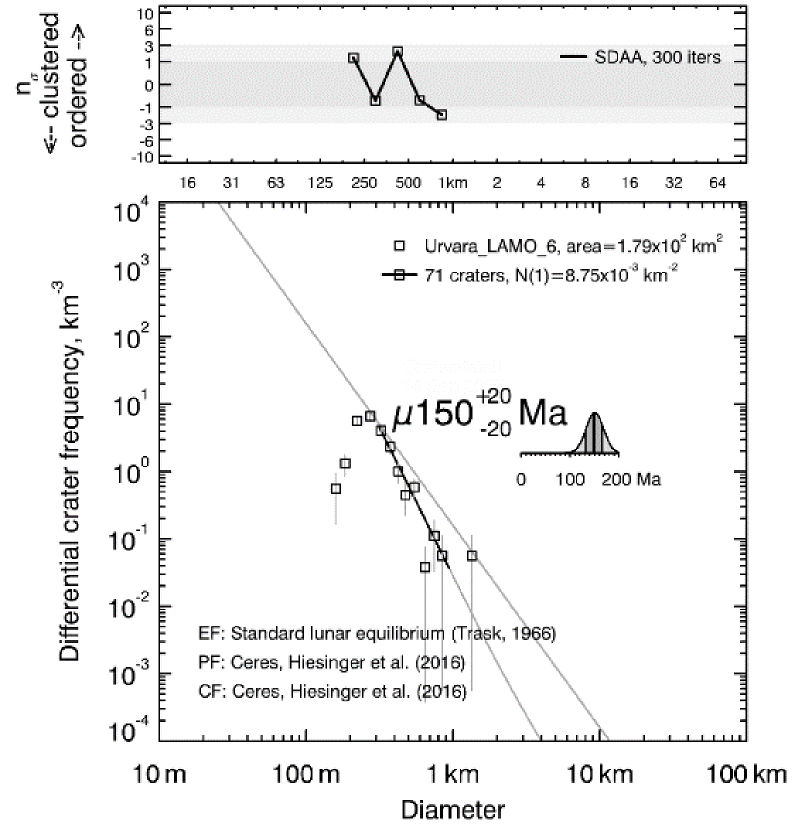

n_σ
<-- clustered
ordered ->
SDAA, 300 iters
16 31 63 125 250 500 1km 2 4 8 16 32 64
Urvara_LAMO_6, area=1.79x10² km²
71 craters, N(1)=8.75x10⁻³ km⁻²
μ150 +20 -20 Ma
0 100 200 Ma
Differential crater frequency, km⁻³
EF: Standard lunar equilibrium (Trask, 1966)
PF: Ceres, Hiesinger et al. (2016)
CF: Ceres, Hiesinger et al. (2016)
10 m 100 m 1 km 10 km 100 km
Diameter

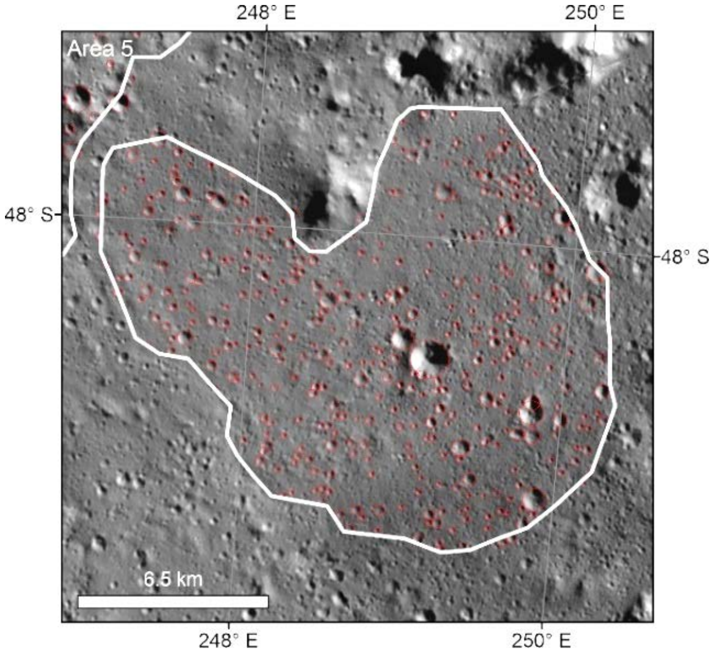

248° E
250° E
Area 5
48° S
48° S
6.5 km
248° E
250° E

Area 7

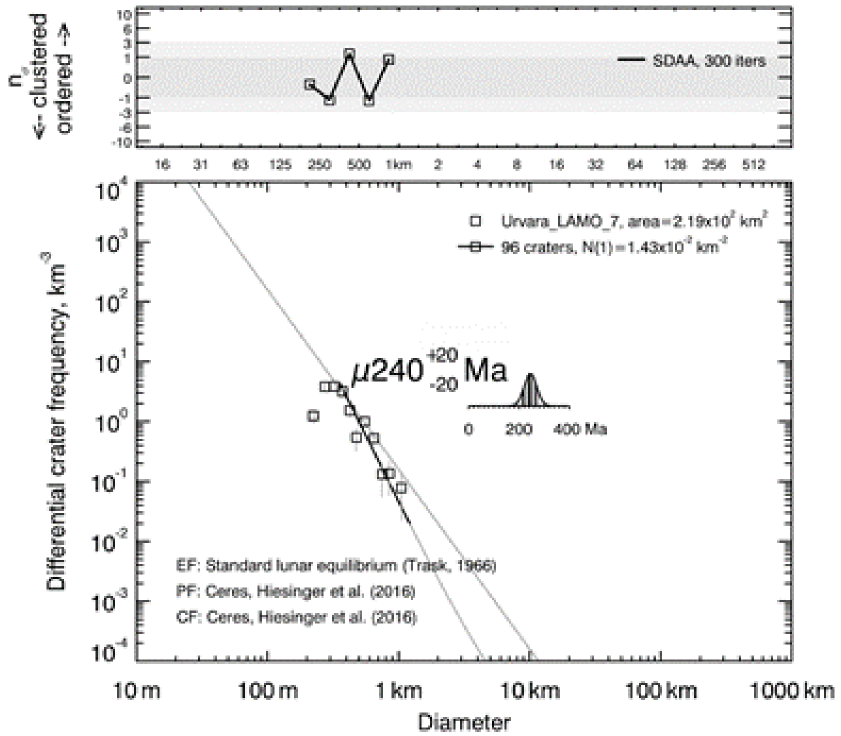

n_σ
<- clustered ordered ->
SDAA, 300 iters
Urvara_LAMO_7, area=2.19x10² km²
96 craters, N(1)=1.43x10⁻¹ km⁻²
μ240 +20 -20 Ma
0 200 400 Ma
Differential crater frequency, km⁻³
EF: Standard lunar equilibrium (Trask, 1966)
PF: Ceres, Hiesinger et al. (2016)
CF: Ceres, Hiesinger et al. (2016)
10 m
100 m
1 km
10 km
100 km
1000 km
Diameter

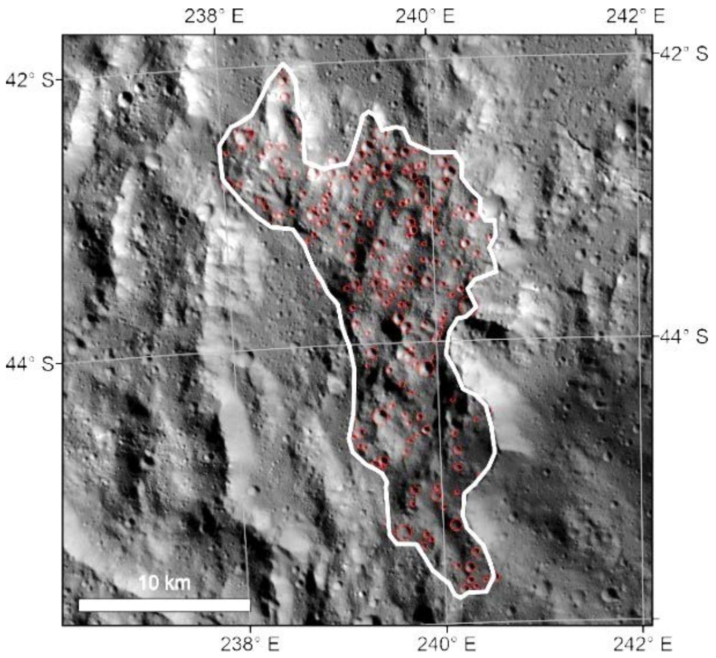

238° E
240° E
242° E
42° S
44° S
10 km

Area 8

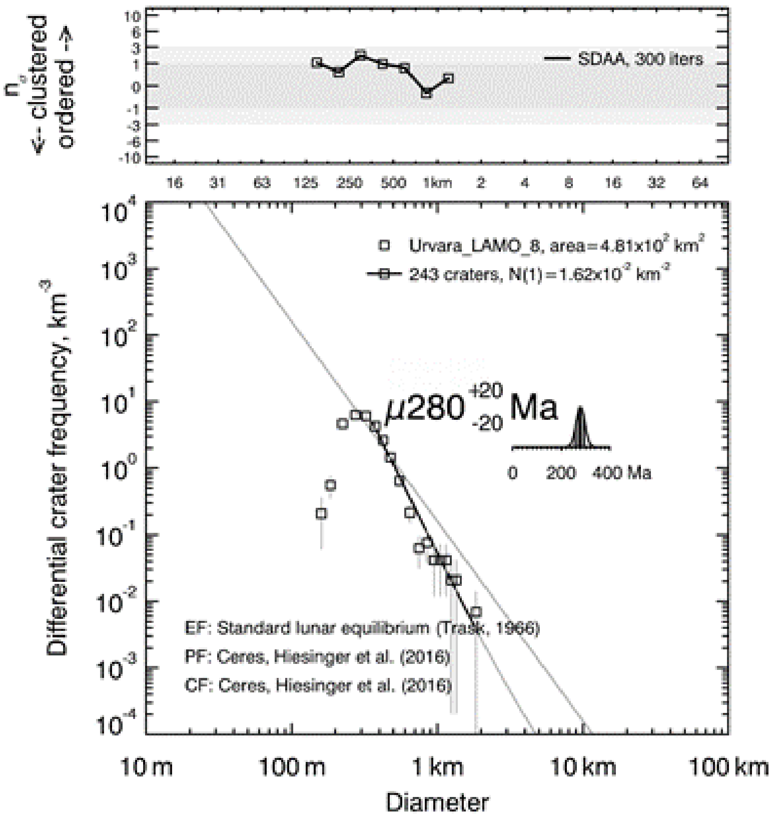

n
<-- clustered
ordered ->
SDAA, 300 iters
16 31 63 125 250 500 1km 2 4 8 16 32 64
Urvara_LAMO_8, area=4.81x10^{2} km^{2}
243 craters, N(1)=1.62x10^{-2} km^{-2}
μ280 $^{+20}_{-20}$ Ma
0 200 400 Ma
Differential crater frequency, km^{-3}
EF: Standard lunar equilibrium (Trask, 1966)
PF: Ceres, Hiesinger et al. (2016)
CF: Ceres, Hiesinger et al. (2016)
10 m 100 m 1 km 10 km 100 km
Diameter

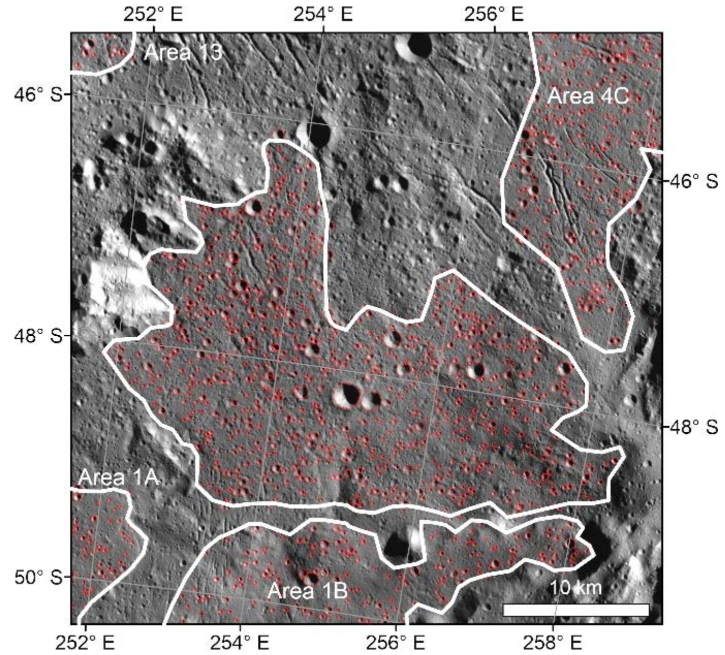

252° E
254° E
256° E
Area 13
Area 4C
46° S
48° S
Area 1A
Area 1B
50° S
10 km
258° E

Area 9

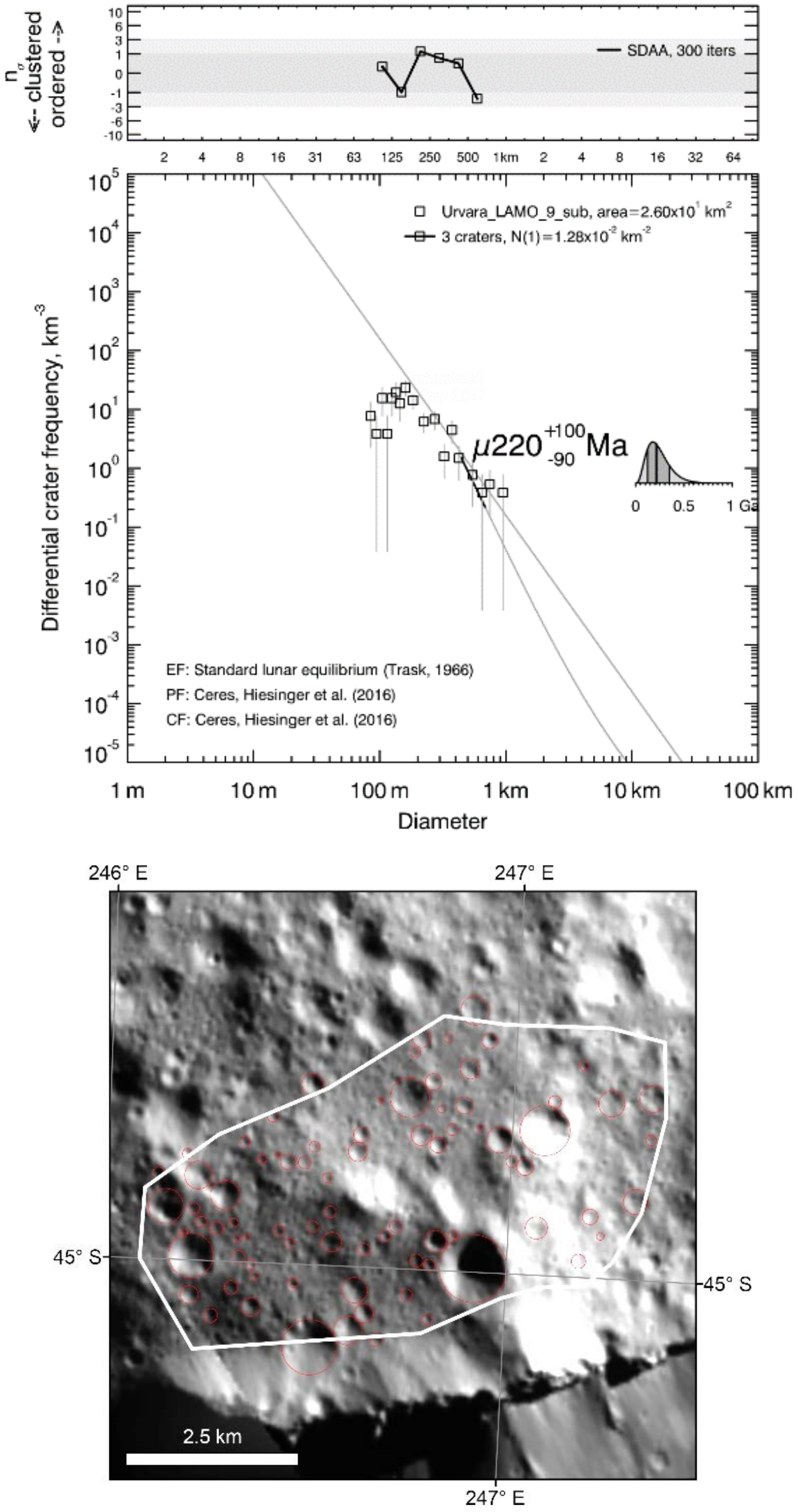

SDAA, 300 iters
n_σ
<-- clustered ordered -->
Urvara_LAMO_9_sub, area=2.60x10^1 km^2
3 craters, N(1)=1.28x10^-2 km^-2
μ220 +100 -90 Ma
0 0.5 1 Ga
Differential crater frequency, km^-3
EF: Standard lunar equilibrium (Trask, 1966)
PF: Ceres, Hiesinger et al. (2016)
CF: Ceres, Hiesinger et al. (2016)
Diameter
1 m
10 m
100 m
1 km
10 km
100 km
246° E
247° E
45° S
2.5 km

Area 10

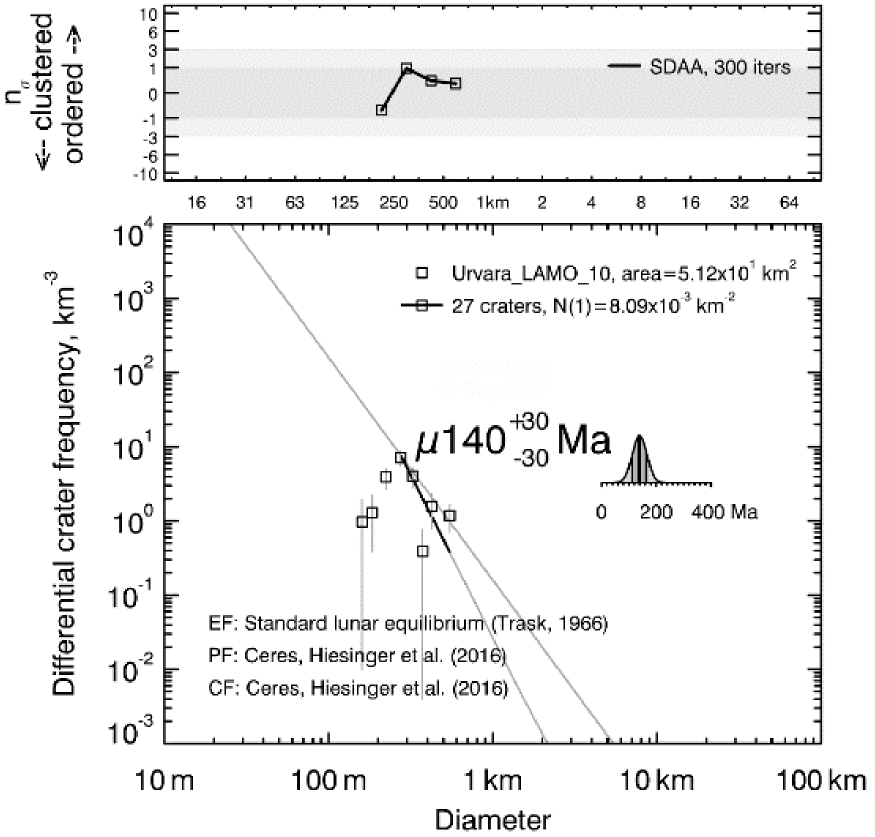

n σ <-- clustered ordered -->
SDAA, 300 iters
Differential crater frequency, km^-3
Urvara_LAMO_10, area=5.12x10^1 km^2
27 craters, N(1)=8.09x10^-3 km^-2
μ140 +30 -30 Ma
0 200 400 Ma
EF: Standard lunar equilibrium (Trask, 1966)
PF: Ceres, Hiesinger et al. (2016)
CF: Ceres, Hiesinger et al. (2016)
10 m
100 m
1 km
10 km
100 km
Diameter

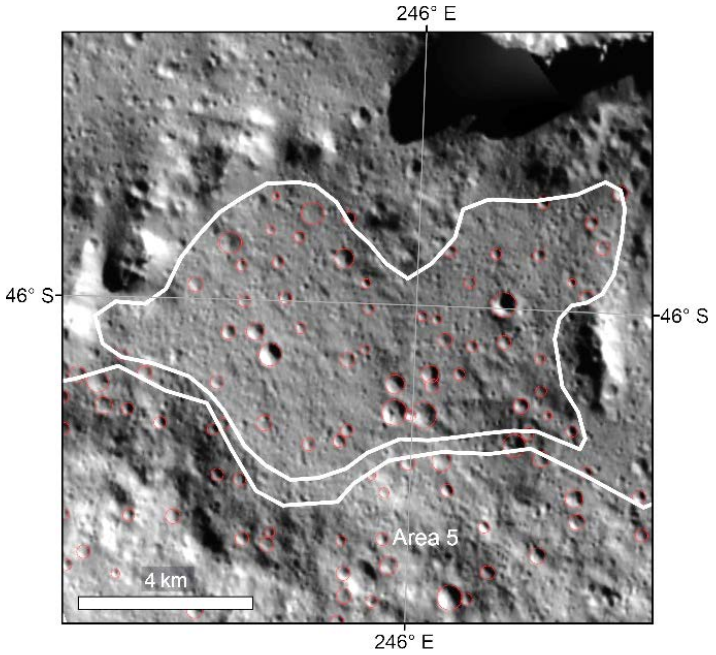

246° E
46° S
46° S
Area 5
4 km
246° E

Area 11

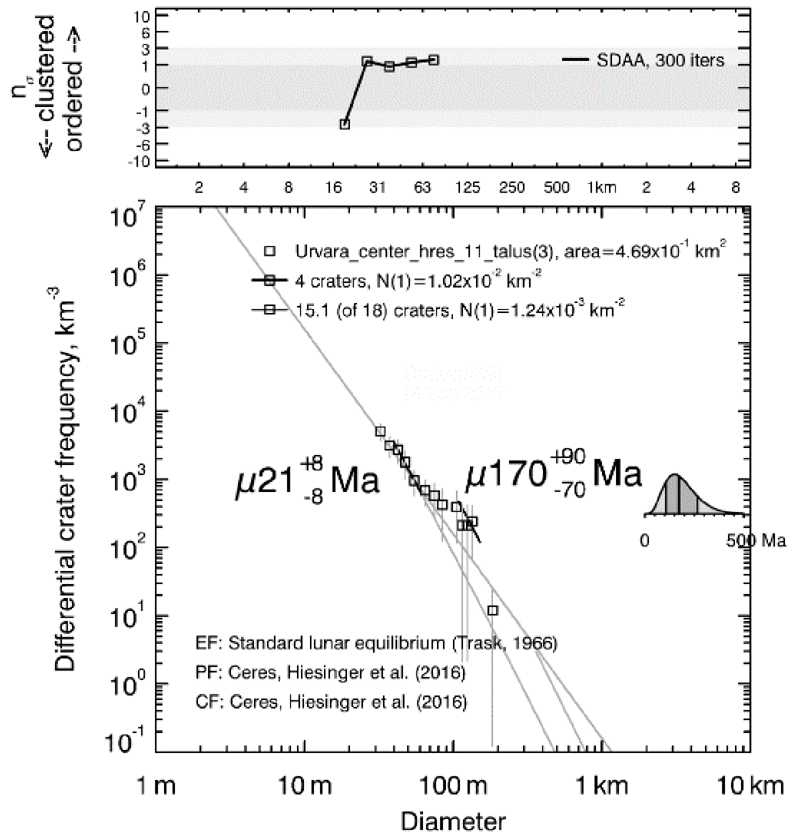

<-- clustered ordered -->
SDAA, 300 iters
Urvara_center_hres_11_talus(3), area=4.69x10^-1 km^2
4 craters, N(1)=1.02x10^-2 km^-2
15.1 (of 18) craters, N(1)=1.24x10^-3 km^-2
μ21 +8 -8 Ma
μ170 +90 -70 Ma
0
500 Ma
EF: Standard lunar equilibrium (Trask, 1966)
PF: Ceres, Hiesinger et al. (2016)
CF: Ceres, Hiesinger et al. (2016)
Differential crater frequency, km^-3
1 m
10 m
100 m
1 km
10 km
Diameter

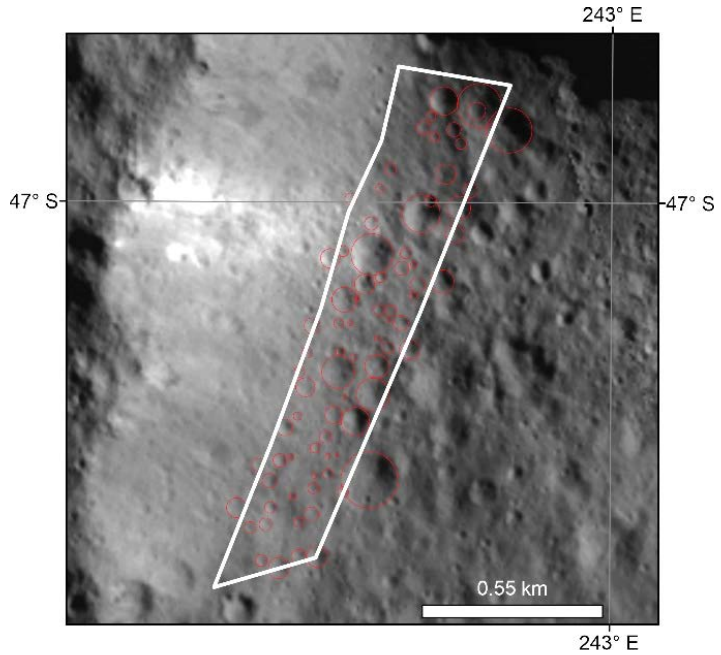

243° E
47° S
47° S
0.55 km
243° E

Area 13

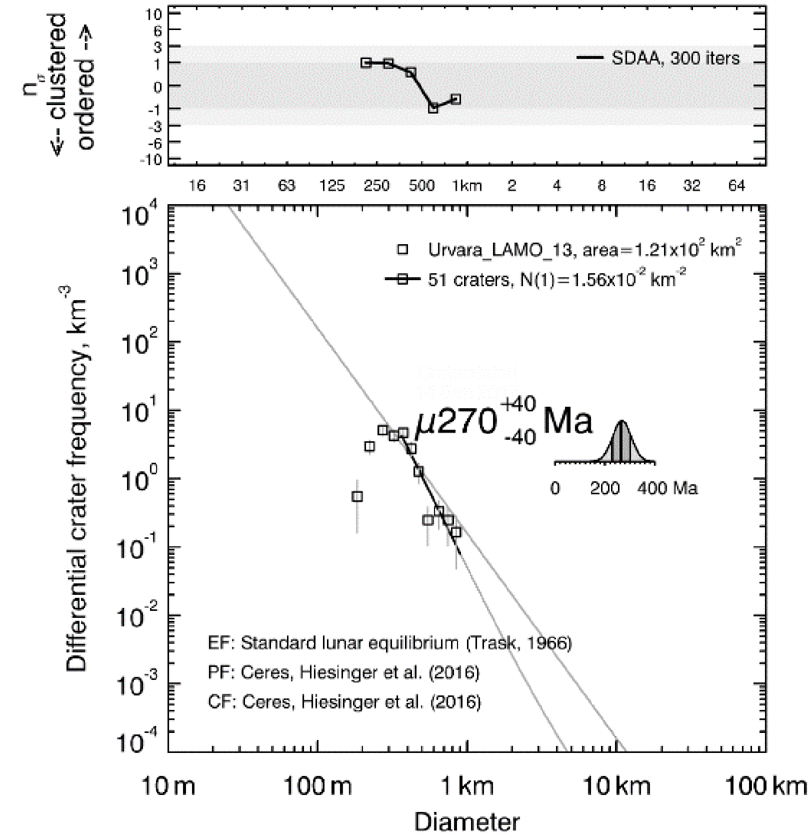

n_σ
<-- clustered
ordered -->
SDAA, 300 iters
10
6
3
1
0
-1
-3
-6
-10
16
31
63
125
250
500
1km
2
4
8
16
32
64
Urvara_LAMO_13, area=1.21x10² km²
51 craters, N(1)=1.56x10⁻² km⁻²
μ270 +40 -40 Ma
0
200
400 Ma
Differential crater frequency, km⁻³
EF: Standard lunar equilibrium (Trask, 1966)
PF: Ceres, Hiesinger et al. (2016)
CF: Ceres, Hiesinger et al. (2016)
10 m
100 m
1 km
10 km
100 km
Diameter

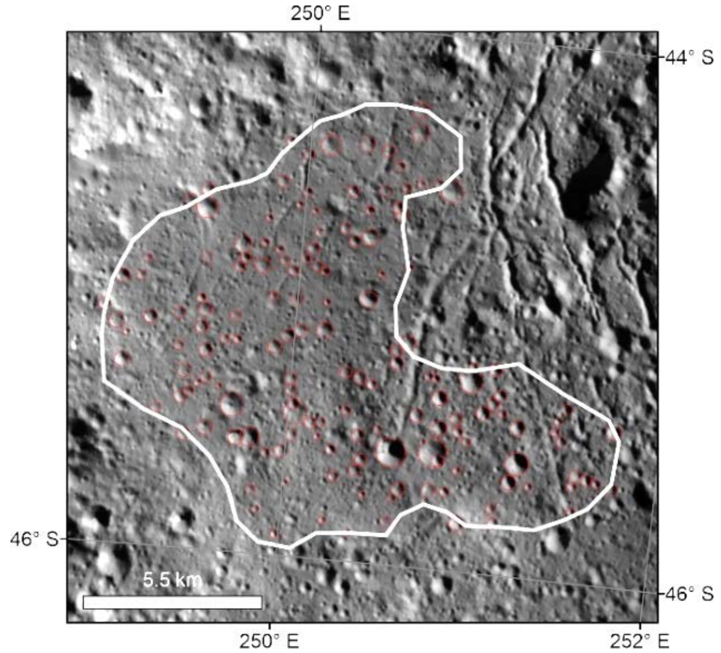

250° E
44° S
46° S
5.5 km
46° S
250° E
252° E

Area 14

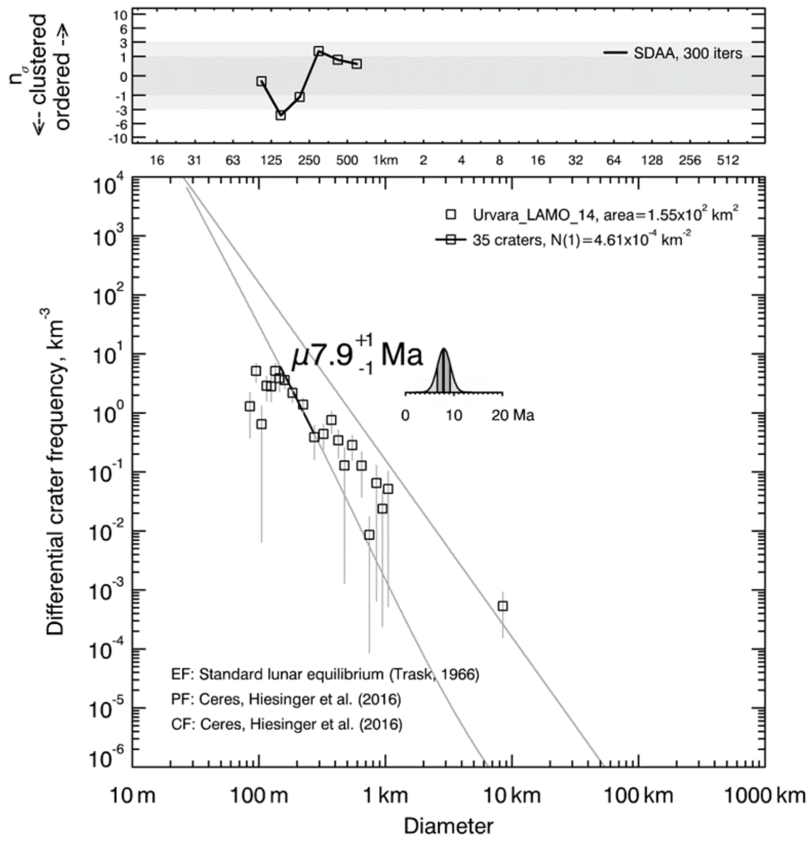

n_σ
<- clustered
ordered ->
SDAA, 300 iters
16 31 63 125 250 500 1km 2 4 8 16 32 64 128 256 512
Urvara_LAMO_14, area=1.55x10² km²
35 craters, N(1)=4.61x10⁻⁴ km⁻²
μ7.9 +1 -1 Ma
0 10 20 Ma
Differential crater frequency, km⁻³
EF: Standard lunar equilibrium (Trask, 1966)
PF: Ceres, Hiesinger et al. (2016)
CF: Ceres, Hiesinger et al. (2016)
10 m 100 m 1 km 10 km 100 km 1000 km
Diameter

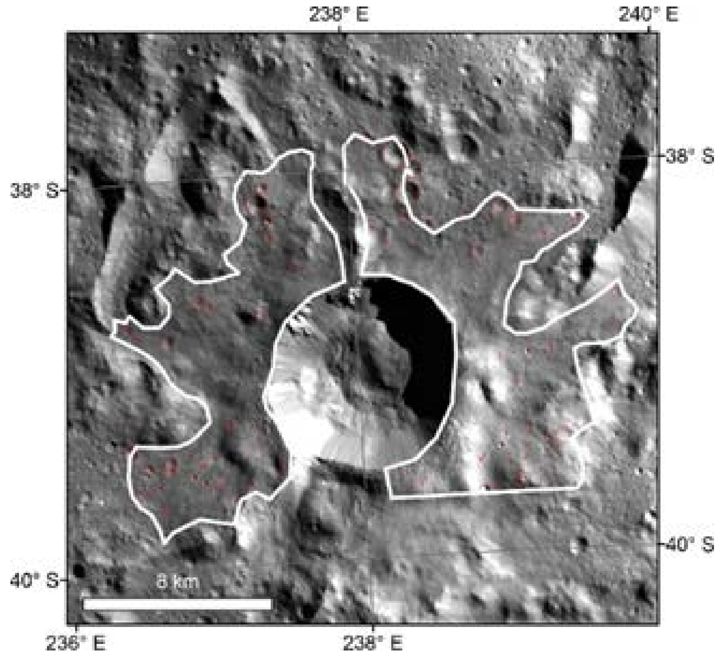

238° E
240° E
38° S
38° S
40° S
40° S
8 km
236° E
238° E

Area 15

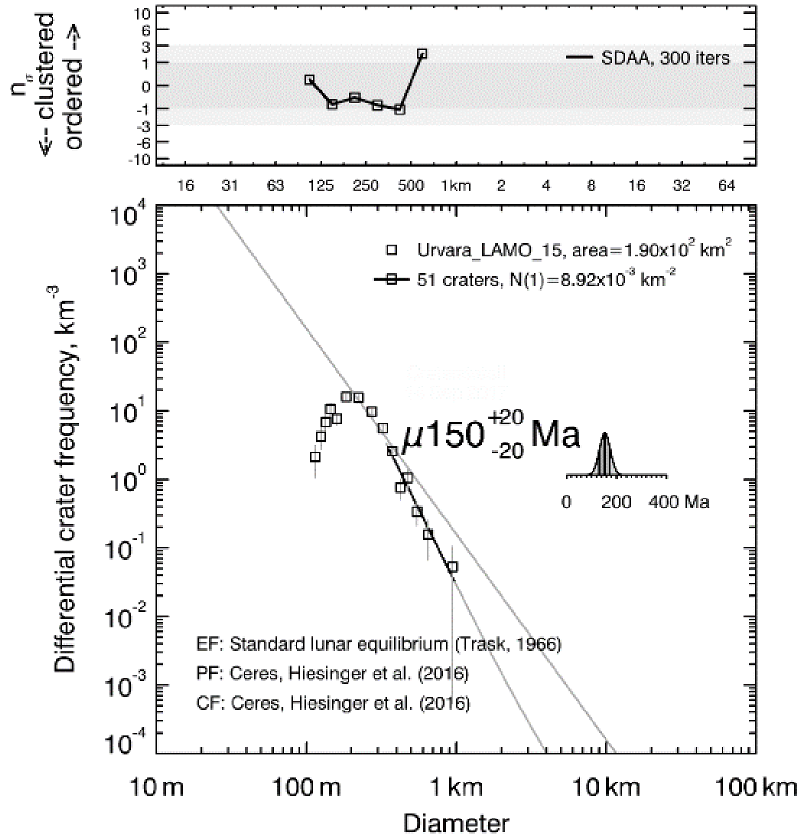

SDAA, 300 iters
Urvara_LAMO_15, area=1.90x10^2 km^2
51 craters, N(1)=8.92x10^-3 km^-2
μ150 +20 -20 Ma
0 200 400 Ma
EF: Standard lunar equilibrium (Trask, 1966)
PF: Ceres, Hiesinger et al. (2016)
CF: Ceres, Hiesinger et al. (2016)
Differential crater frequency, km^-3
Diameter

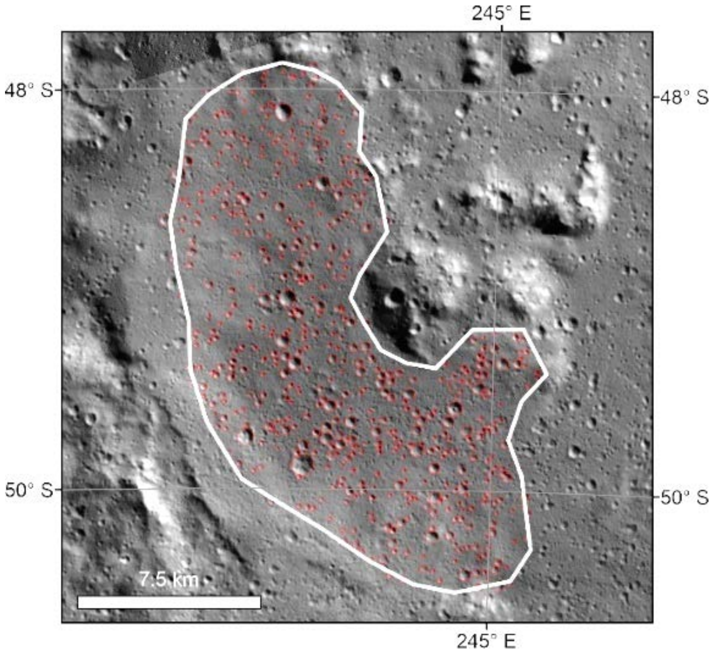

245° E
48° S
50° S
7.5 km

Area 16

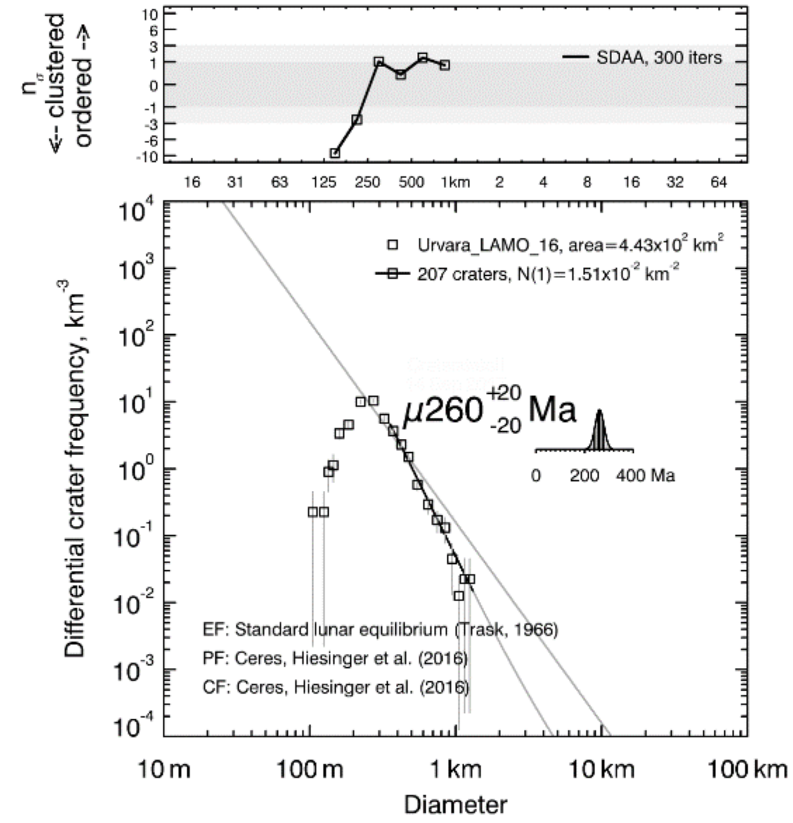

n_σ
<-- clustered
ordered -->
SDAA, 300 iters
16 31 63 125 250 500 1km 2 4 8 16 32 64
Urvara_LAMO_16, area=4.43x10^2 km^2
207 craters, N(1)=1.51x10^-2 km^-2
μ260 +20 -20 Ma
0 200 400 Ma
EF: Standard lunar equilibrium (Trask, 1966)
PF: Ceres, Hiesinger et al. (2016)
CF: Ceres, Hiesinger et al. (2016)
Differential crater frequency, km^-3
10 m 100 m 1 km 10 km 100 km
Diameter

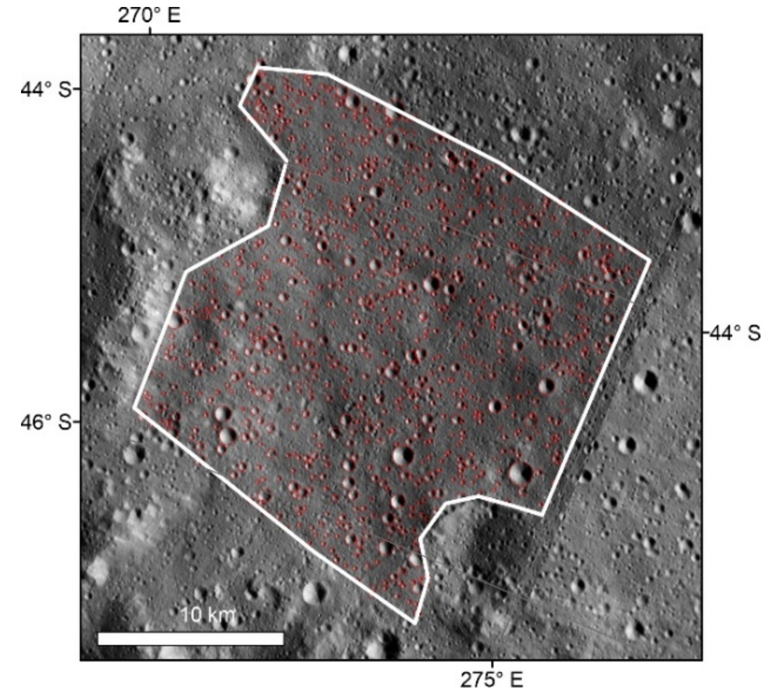

270° E
44° S
44° S
46° S
10 km
275° E

Area 17

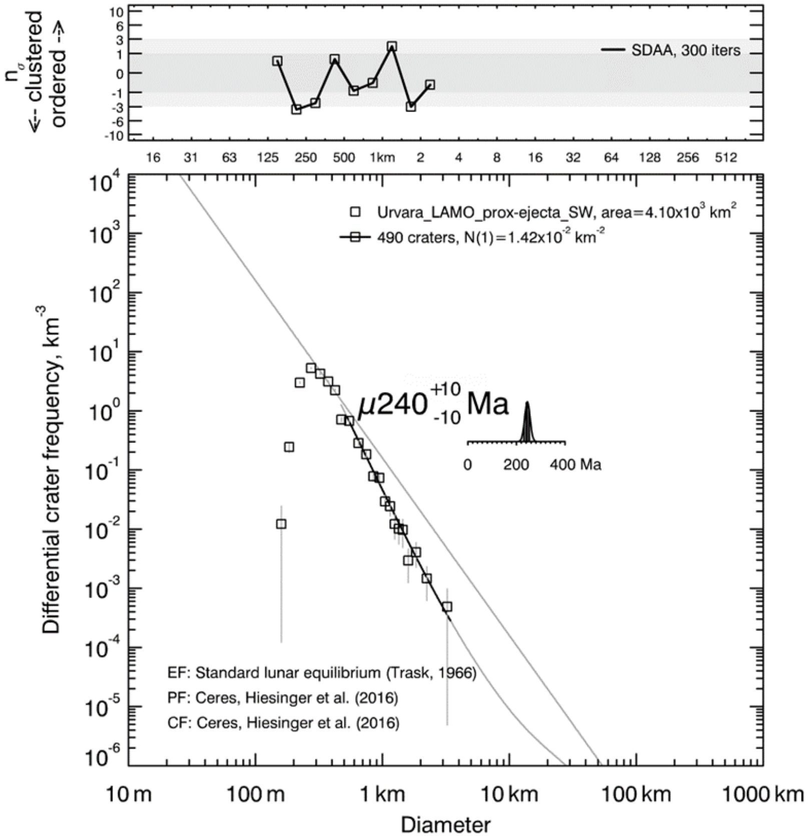

n_σ
<-- clustered
ordered ->
SDAA, 300 iters
Urvara_LAMO_prox-ejecta_SW, area=4.10x10^3 km^2
490 craters, N(1)=1.42x10^-2 km^-2
μ240 +10 -10 Ma
0 200 400 Ma
Differential crater frequency, km^-3
EF: Standard lunar equilibrium (Trask, 1966)
PF: Ceres, Hiesinger et al. (2016)
CF: Ceres, Hiesinger et al. (2016)
10 m
100 m
1 km
10 km
100 km
1000 km
Diameter

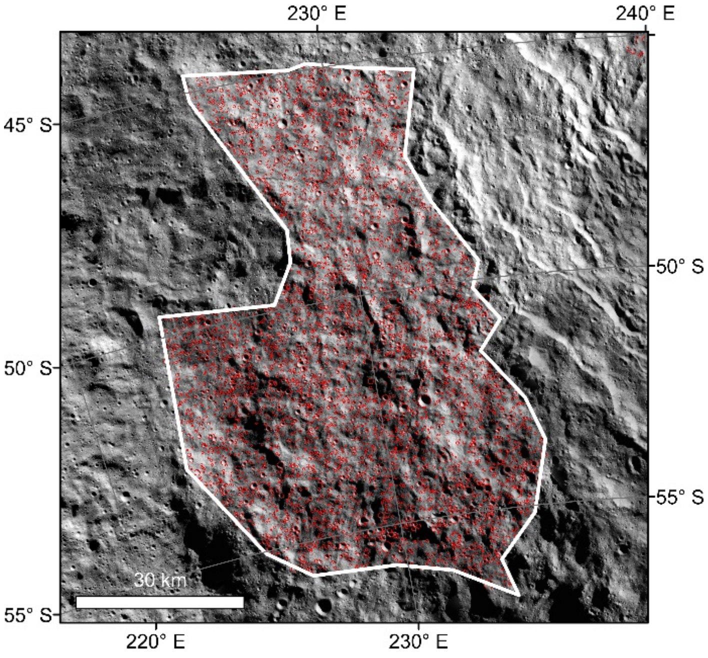

230° E
240° E
45° S
50° S
50° S
55° S
30 km
55° S
220° E
230° E

References

[1] Park, R. S. et al. High-resolution shape model of Ceres from stereophotoclinometry using Dawn Imaging Data. *Icarus* **319**, 812–827 (2019). https://doi.org/10.1016/j.icarus.2018.10.024

[2] Beyer et al. 1999 The Ames Stereo Pipeline:NASA's Open Source Automated Stereogrammetry SoftwareVersion 2.6.2_postIntelligent Robotics GroupNASA Ames Research User Manual, (2019). Centerstereo-pipeline-owner@lists.nasa.gov

[3] Crown, D. A. et al. Geologic mapping of the Urvara and Yalode Quadrangles of Ceres: *Icarus* **316,** 167–190(2018). ISSN 0019-1035, https://doi.org/10.1016/j.icarus.2017.08.004

[4] Wyrick, D. et al. Distribution, morphology, and origins of Martian pit crater chains. *Journal of Geophysical Research: Planets* **109,** E6 (2004). https://doi.org/10.1029/2004JE002240

[5] Ferrill, D.A. et al. Dilational fault slip and pit chain formation on Mars. *GSA Today* **14,** 10, 4–12. (2004). https://doi.org/10.1130/1052-5173(2004)014<4:DFSAPC>2.0.CO;2

[6] Bardintzeff, J., McBirney, A. R. Basaltic lavas, in Volcanology, 2nd ed., pp. 69–73, Jones and Bartlett Publishers, Boston, Mass. (2000). https://doi.org/10.1007/s00445-011-0551-3

[7] Mège, D. Y. et al. Collapse features and narrow grabens on Mars and Venus: Dike emplacement and deflation of underlying magma chamber (abstract), Proc. Lunar Planet. Sci. Conf. 31st, 1854. (2000). https://www.lpi.usra.edu/meetings/lpsc2000/pdf/1854.pdf

[8] Mège, D. et al. Volcanic rifting at Martian grabens. *Journal of Geophysical Research* **108,** E5, 5044 (2003). https://doi.org/10.1029/2002JE001852

[9] Spencer, J., Fanale, F. New models for the origin of Valles Marineris closed depressions, *Journal of Geophysical Research* **95**, 14,301–14,313 (1990). https://doi.org/10.1029/JB095iB09p14301

[10] Handy M. R. et al. Continental Fault Structure and Rheology from the Frictional-to-Viscous Transition Downward, Tectonic Faults: Agents of Change on a Dynamic Earth, MIT Press, 139–182 (2007).

[11] Otto, K. A. et al. Ceres Crater degradation inferred from concentric fracturing. *J. Geophys. Res. Planets* **124**, 1188–1203 (2019). https://doi.org/10.1029/ 2018JE005660

[12] Bland, M.T., Predicted crater morphologies on Ceres: Probing internal structure and evolution. *Icarus* **226,** 1, 510–521. https://doi.org/10.1016/j.icarus.2013.05.037

[13] Bowling, T. J. et al., Post-impact thermal structure and cooling timescales of Occator crater on asteroid 1 Ceres. *Icarus* **320**, 110–118. (2019), https://doi.org/10.1016/j.icarus.2018.08.028
